# Evaluating Digital Program Support for the Physical Activity 4 Everyone (PA4E1) School Program: Mixed Methods Study

**DOI:** 10.2196/26690

**Published:** 2021-07-26

**Authors:** Matthew Mclaughlin, Jed Duff, Tom McKenzie, Elizabeth Campbell, Rachel Sutherland, John Wiggers, Luke Wolfenden

**Affiliations:** 1 School of Medicine and Public Health University of Newcastle Callaghan Australia; 2 Hunter New England Population Health Wallsend Australia; 3 Hunter Medical Research Institute New Lambton Heights Australia; 4 Priority Research Centre for Heath Behaviour University of Newcastle Callaghan Australia; 5 School of Nursing and Midwifery University of Newcastle Callaghan Australia; 6 Centre for Healthcare Transformation Queensland University of Technology Kelvin Grove Australia

**Keywords:** process evaluation, engagement, think-aloud methodology, mixed methods, physical activity, website, digital health intervention, implementation support, delivery mode, scale-up

## Abstract

**Background:**

Effectively scaled-up physical activity interventions are urgently needed to address the high prevalence of physical inactivity. To facilitate scale-up of an efficacious school-based physical activity program (Physical Activity 4 Everyone [PA4E1]), provision of implementation support to physical education (PE) teachers was adapted from face-to-face and paper-based delivery modes to partial delivery via a website. A lack of engagement (usage and subjective experience) with digital delivery modes, including websites, may in part explain the typical reduction in effectiveness of scaled-up interventions that use digital delivery modes. A process evaluation focused on the PA4E1 website was undertaken.

**Objective:**

The 2 objectives were to (1) describe the usage of the PA4E1 program website by in-school champions (PE teachers leading the program within their schools) and PE teachers using quantitative methods; (2) examine the usage, subjective experience, and usability of the PA4E1 program website from the perspective of in-school champions using mixed methods.

**Methods:**

The first objective used website usage data collected across all users (n=273) throughout the 9 school terms of the PA4E1 implementation support. The 4 usage measures were sessions, page views, average session duration, and downloads. Descriptive statistics were calculated and explored across the duration of the 26-month program. The second objective used mixed methods, triangulating data from the first objective with data from a think-aloud survey and usability test completed by in-school champions (n=13) at 12 months. Qualitative data were analyzed thematically alongside descriptive statistics from the quantitative data in a triangulation matrix, generating cross-cutting themes using the “following a thread” approach.

**Results:**

For the first objective, in-school champions averaged 48.0 sessions per user, PE teachers 5.8 sessions. PE teacher sessions were of longer duration (10.5 vs 7.6 minutes) and included more page views (5.4 vs 3.4). The results from the mixed methods analysis for the second objective found 9 themes and 2 meta-themes. The first meta-theme indicated that the website was an acceptable and appropriate delivery mode, and usability of the website was high. The second meta-theme found that the website content was acceptable and appropriate, and identified specific suggestions for improvement.

**Conclusions:**

Digital health interventions targeting physical activity often experience issues of lack of user engagement. By contrast, the findings from both the quantitative and mixed methods analyses indicate high usage and overall acceptability and appropriateness of the PA4E1 website to school teachers. The findings support the value of the website within a multidelivery mode implementation intervention to support schools to implement physical activity promoting practices. The analysis identified suggested intervention refinements, which may be adopted for future iterations and further scale-up of the PA4E1 program.

**Trial Registration:**

Australian New Zealand Clinical Trials Registry ACTRN12617000681358; https://www.anzctr.org.au/Trial/Registration/TrialReview.aspx?id=372870

## Introduction

### Background

As much as 1 in 4 (25%) adults and 4 in 5 (80%) adolescents do not meet the global recommendations for aerobic exercise and are therefore at increased risk of noncommunicable diseases and premature mortality [1,2]. Scalable programs with proven effectiveness are urgently needed to increase population physical activity, including those in the school setting, as outlined in the Global Action Plan on Physical Activity 2018-2030 [3].

Many efficacious health interventions exist [4], but scaling-up efficacious interventions with proven health benefits is important to ensure the population health benefit of such interventions can be realized [5]. Digital delivery modes are often used to facilitate scale-up of efficacious interventions, as they have the potential to achieve considerable reach at relatively low cost compared with traditional modes of program delivery [4]. A recent systematic review of scaled-up trials of obesity-prevention interventions found that the main adaptations to interventions to facilitate their scale-up were changes in the modes of delivery [4]. Specifically, websites or other digital delivery modes were commonly added or replaced face-to-face components, for example, to provide program resources and training for teachers or clinicians in program delivery [4]. Research examining digital delivery modes such as websites, however, often report poor usage and engagement with such technologies [6-8].

A lack of engagement with digital technologies [4] may, in part, explain the modest impact of physical activity interventions that have relied on such technologies to support population-wide scale-up [4,6,9]. A recent meta-analysis of digital health interventions targeting physical activity found that higher usage engagement is associated with targeted behavior changes [10]. Perski et al [9] have conceptualized engagement with digital health interventions to include both amount, duration and depth of *usage*, and user *subjective experience*, characterized by attention, interest, and affect [9]. Systematic reviews of the human–computer interaction literature suggest that good usability (ie, functionality and efficiency of the digital application) and subjective experience engagement (ie, users attention, interest, and affect) are important drivers of reducing attrition and increasing usage of digital applications [9,11].

Process evaluations are recommended as part of comprehensive evaluations of all randomized trials to aid the interpretation of trial findings and to better understand and explain why an intervention has or has not worked [12]. Process evaluations may be particularly important as part of trials of interventions that have been scaled-up, given the limited research and insight scientists currently have regarding scale-up processes. However, detailed process evaluations are seldom undertaken. For example, a recent systematic review of school-based physical activity interventions found that just 4 of the 17 trials included in the review had conducted a process evaluation [13]. Further, of those undertaken in the secondary school setting, few have used mixed methods [14-17] and many have focused on a narrow range of quantitative process outcomes (eg, fidelity, reach, dose) [18-21]. Perhaps most importantly for those interested in the application of digital technologies to support scale-up, none of the trials identified in the review [4], or in reviews of the scale-up literature generally, have undertaken a process evaluation examining the key dimensions of subjective experience and usage of digital components employed to deliver implementation support strategies.

Given the lack of process evaluations focused on the digital delivery mode of interventions utilizing multiple delivery modes, this study sought to address this evidence gap by conducting a process evaluation of the digital delivery mode used to support scale-up of an effective health intervention. This study addresses the scale-up of Physical Activity 4 Everyone (PA4E1), a secondary school–based physical activity program that included a website to support the delivery of implementation support strategies to school teachers (physical education [PE] teachers and in-school champions [PE teachers leading the program within their schools]). The objectives of this study are below, followed by an overview of the PA4E1 program and evaluation trials, which provide more detailed context for this study.

1. To describe the usage of the PA4E1 program website by in-school champions and PE teachers using quantitative methods.
2. Examine the usage, subjective experience, and usability of the PA4E1 program website from the perspective of in-school champions using mixed methods.

### Physical Activity 4 Everyone (PA4E1)

School practices, programs, and policies can support adolescents to be physically active and are recommended by the World Health Organization and governments internationally through whole-school approaches [3,22-24]. One school program that has been shown to assist schools (PE teachers, principals) to support their students to become more physically active is PA4E1 [25-27].

### PA4E1 Prescale: Efficacy Trial

PA4E1 was first trialed from 2012 to 2014 as a 2-year randomized controlled efficacy trial in low-socioeconomic Australian secondary schools [25]. PA4E1 had positive effects on student physical activity and unhealthy weight gain [25-31]. The PA4E1 program consisted of 7 physical activity practices, and 6 implementation support strategies.

### PA4E1 Postscale (This Study): Type III Hybrid Implementation–Effectiveness Trial

**Figure 1:**
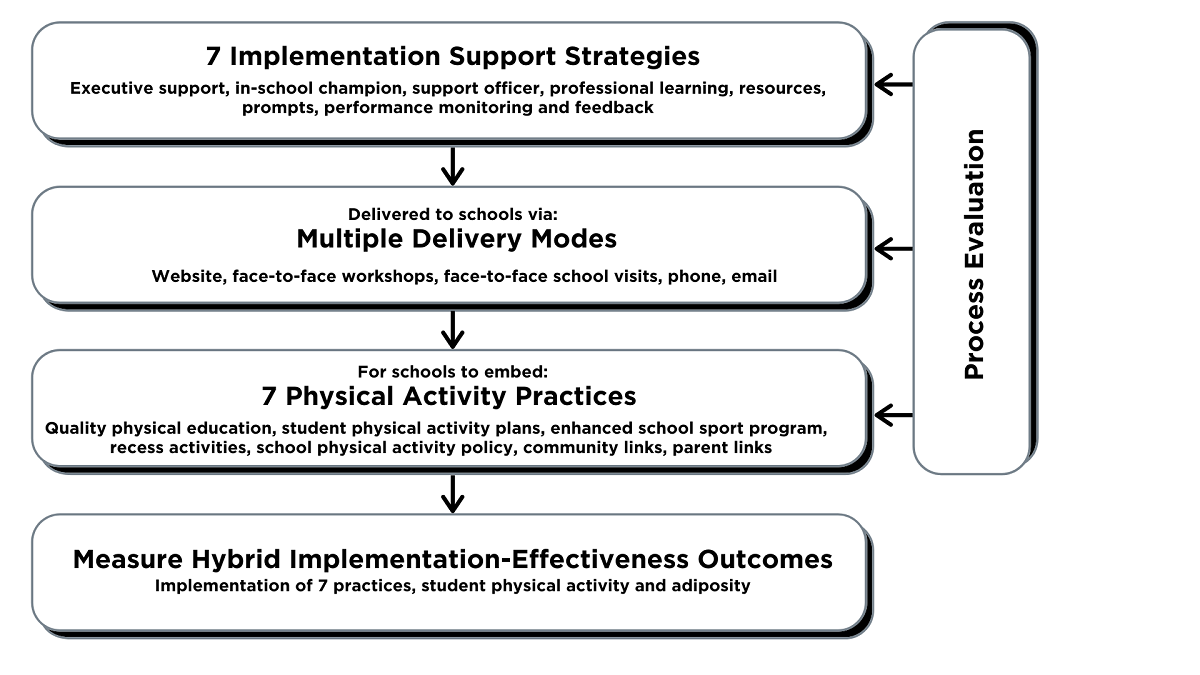
The PA4E1 scale-up trial logic model, expanded from [28]. PA4E1: Physical Activity 4 Everyone.

The program was scaled-up for delivery in more schools across a larger geographic area, utilizing a website optimized for desktop, mobile, and tablet devices, to support the delivery of implementation support strategies to schools. The PA4E1 scale-up trial was a type III hybrid implementation–effectiveness cluster randomized controlled trial [29]. The trial involved 49 schools, 24 allocated to the program (intervention) group. An outline of the logic for this trial is shown in Figure 1. Program schools were offered 7 implementation support strategies to support their adoption of 7 physical activity practices, supporting school students to become more physically active. Details of the physical activity practices and implementation support strategies (as well as additional information on the timing of the implementation support strategies) are provided in Multimedia Appendix 1. Adaptations for scale-up were made to both the physical activity practices and the implementation support strategies, as detailed elsewhere [29]. The main change relevant to this study was the introduction of a digital delivery mode, a website, for the provision of program implementation support. Multimedia Appendix 1 (Table S1) shows how the website was used within the 7 implementation support strategies (n=23 substrategies). To summarize, a password-protected program website replaced face-to-face and paper-based delivery modes to part-deliver teacher professional learning to all PE teachers in participating schools; provide program resources for in-school champions and PE teachers; prompt in-school champions and PE teachers to implement PA4E1; monitor schools’ performance on meeting practice implementation milestones in each school term via an in-school champion–completed termly survey; and provide feedback to school stakeholders (in-school champions, principals) based on their termly survey results [25,29].

The primary trial outcome was uptake of physical activity practices by schools at study midpoint (12 months) and 24 months. At 12 months the trial’s primary outcome (proportion of schools adopting at least four of the seven physical activity practices) was significant, with more schools implementing 4 of the 7 practices in the program group (16/24, 67%) than in the control group (1/25, 4%; *P*<.001) [32]. Further process evaluation outcomes will be reported elsewhere, in line with the process evaluation protocol [28].

## Methods

The methods are reported by objective.

### Objective 1: Describe the Usage of the PA4E1 Program Website by In-School Champions and PE Teachers Using Quantitative Methods

#### Sampling

Usage data were tracked for the entire study period (October 2017 to December 2019; 9 school terms) for all users of the PA4E1 website who had a registered account with the program website, including in-school champions, PE teachers, principals, school administration staff, and nonschool staff (support officers and the PA4E1 research team). At the start of the program, all users were provided a registration link via email to register for the website. During the program, new school staff were provided a link to register for the website.

#### Data Collection

Usage data were collected via Google Analytics throughout the 9 terms of the program (26 months, October 9, 2017, to December 20, 2019). Prior to March 29, 2018 (term 3), data could not be analyzed by website user type; only overall usage could be captured. In order to further understand the different types of users, a “custom view” was applied in Google Analytics from this date to prospectively segregate different school user types (ie, in-school champions, PE teachers).

#### Measures

The post hoc analysis method described by the Analyzing and Measuring Usage and Engagement Data (AMUsED) framework [33] was used to guide the selection of usage variables to report (Multimedia Appendix 2). Google Analytics was used to track 4 variables for the entire study period (Textbox 1).

**Variables tracked using Google Analytics.**

**Sessions**

Similar to a “login,” sessions are defined as a group of user interactions (eg, page views, downloads) that occur within a single period, specifically a session ends after 30 minutes of inactivity or if users leave and revisit the website. Sessions were chosen as a better measure than “logins” for the PA4E1 website as users remained logged in for a rolling period of 30 days, whereby users were only logged out following 30 days of inactivity.

**Page Views**

Page view is a count of total visits to each page of the website. If a user clicks reload after reaching the page, this was counted as an additional page view. If a user navigated to a different page and then returned to the original page, a second page view was recorded.

**Average Session Duration**

The length of time of a session from the first click to the last, excluding the inactive period of a session immediately following the last click.

**Downloads**

A count of the total number of downloads of resources from the resources page of the website. Users had to click onto the particular resource to be counted as a download.

#### Data Analysis

Data were first downloaded from Google Analytics for the entire study period. Descriptive statistics were produced in SAS software [34] for all users for the entire study period and for each type of user from March 29, 2018. Mean and SD were calculated for each measure.

### Objective 2: Examine the Usage, Subjective Experience, and Usability of the PA4E1 Program Website From the Perspective of In-School Champions Using Mixed Methods

#### Sampling

All in-school champions (n=24) were invited to complete a “think-aloud” survey and usability test via email after 4 school terms of website use (November 2018) [35]. Participants could complete the survey anytime through until May 2019 (Term 6). All in-school champions were emailed a study information letter and informed that completion of the survey acted as consent to the study. Completion of the survey and usability test was expected to take 25-30 minutes based on piloting. Participants were provided with an AUD $30 (US $23) e-gift card reimbursement for completing the survey.

#### Data Collection

**Figure 2:**
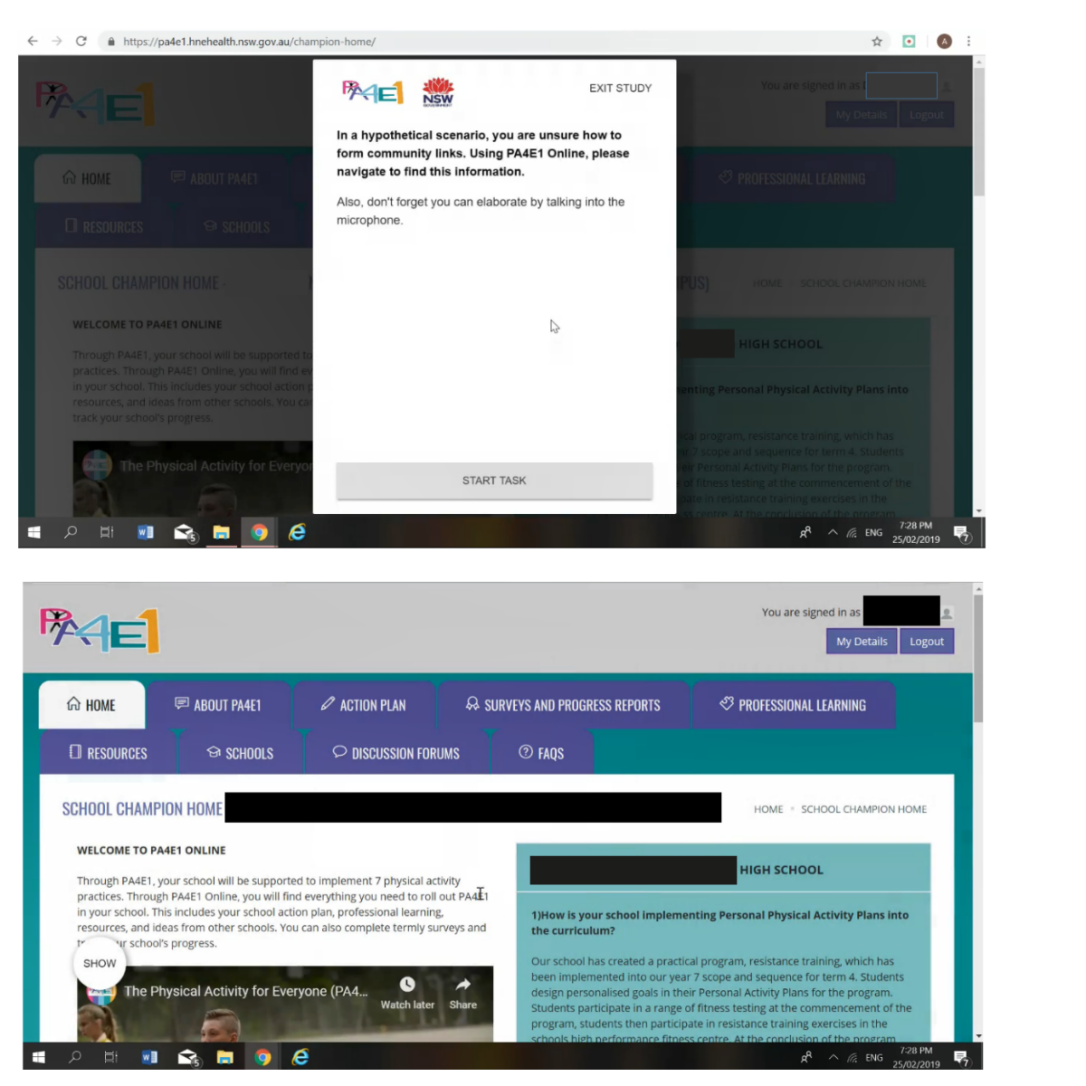
Two screenshots of a participant completing the think-aloud survey and usability test, with the survey imposed on the program website.

The think-aloud survey and usability test was conducted remotely via in-school champions’ own digital devices (ie, in-school champions’ own mobile, laptop, or computers). Loop11 user-testing software was used to conduct the think-aloud survey and usability test [36]. As Figure 2 shows, Loop11 displays a set of questions and tasks imposed upon the website [36]. Throughout the think-aloud survey and usability test, participants were prompted by the Loop 11 platform to verbalize their thoughts while responding online to a series of questions (survey) and tasks (usability test) (Multimedia Appendix 3). The researchers were not present during the study, and participants were invited to complete the study in their own time using a link provided to them via email. This method was chosen to increase the real-world relevance of the findings, as the participants complete the activities in their own setting [9]. As well as the quantitative responses from the survey questions and usability tasks, a video screen capture and microphone audio data were also collected concurrently, and participants were frequently encouraged to explain their responses by “thinking aloud.” A practice question was designed to give participants the opportunity to familiarize themselves with the format of the Loop11 user testing platform and to practice thinking aloud.

#### Measures

Figure 2 shows a screenshot of the think-aloud survey and usability test which is imposed upon the program website. The survey contained 4 validated tools [37-39]. All tools were on a 5-point Likert-scale (1=strongly disagree, 2=disagree, 3=neutral, 4=agree, and 5=strongly agree). The Systems Usability Scale is a 10-item scale to assess usability of the website (scores range 0-100) [37] and comparable data exist to interpret the score derived from the Systems Usability Scale [40]. The second and third tools were the Acceptability of Intervention [39] and Intervention Appropriateness Measure [39], to assess the acceptability and appropriateness of the website, respectively (scores range 0-5). The fourth tool was the long form of the User Engagement Scale [38], which is a 31-item tool split into 4 subscales (Aesthetic appeal; Focused attention; Perceived usability; and Reward; scores range 0-5 for each subscale and overall).

The usability test contained 3 tasks. The average time to task completion and success rate of task completion were recorded within Loop11. Tasks asked participants to navigate to certain sections of the website. The first task asked participants to navigate to the discussion forum, the second to find information on forming community links (see Practice 7 in Table S2 of Multimedia Appendix 1) and the final task asked participants where they would post a lesson observation (see Practice 1 in Table S2 of Multimedia Appendix 1). Participants were encouraged to verbalize their thoughts (think aloud) throughout the 3 tasks.

The survey also contained 12 prompting questions which asked participants to think aloud to verbalize their responses (see Multimedia Appendix 3 for a full list of these questions). The questions asked participants to explain their thoughts toward a particular hypothetical scenario or comment on a particular aspect of the website (eg, usefulness of the website if PA4E1 were to be rolled out state-wide). The questions were used to elicit additional responses from participants. Concurrently, audio data and a video of the participants screen (screen capture) were recorded.

Additional administration data were used to identify usage of other delivery modes for implementation support strategies (Figure 1), including attendance at face-to-face events and contact logs between in-school champions and support officers (data sources have been described in the process evaluation protocol [28]).

### Mixed Method Data Analysis

A mixed methods data analysis was informed by Perski et al’s [9] conceptualization of digital behavior change intervention engagement framework, whereby *delivery* and *content* were considered separately. *Delivery* focuses on themes that emerge relating to the aesthetics/design, challenge, complexity, control features, credibility features, ease of use, familiarity, guidance, interactivity, message tone, mode of delivery, novelty, narrative, personalization, and professional support features of the digital intervention. *Content* focuses on themes that emerge relating to the behavior change techniques (eg, feedback, goal setting, reminders, rewards, self-monitoring, social support features). Themes relating to delivery and content were extracted separately.

All quantitative data were downloaded and analyzed in MS Excel. The quantitative data from the validated tools were scored according to their instructions [37-39] and summary descriptive statistics were produced (mean score [SD]). Task completions were calculated as percentage of successful completions compared with total attempts. Time taken to complete tasks were recorded from the initial click to the final successful click by the participant. Mean time and SD were calculated across all in-school champions for each task.

Audio data were transcribed verbatim and analyzed using inductive thematic analysis [41]. Screen recording data were also downloaded (video) and analyzed concurrently with the audio data, within Nvivo 12 (QSR International) [42], to provide additional context to the audio data (eg, a participant’s cursor location within website).

The quantitative and qualitative data were mixed during analysis using a triangulation matrix. Both data sets were given equal emphasis to address the objective. The descriptive statistics were produced at the same time as the initial codes of the qualitative data. As per the process evaluation protocol [28], the “following the thread” approach was employed to generate hypothesis and questions of the qualitative data from the quantitative data, and vice versa. The findings were combined and compared (triangulated) using a triangulation matrix to assess where findings from one method agreed or partially agreed (convergence), appear to contradict each other (discrepancy or dissonance), or are silent (ie, a theme arises in 1 data set but not in another). The matrix allowed the production of themes and meta-themes (higher-level themes) that combined and compared the qualitative and quantitative data sets [41]. Throughout the entire analysis, MM kept a research journal.

The iterative phases of the mixed method analysis [41] included:

- Familiarizing with the data (both audio and screen capture as well as quantitative data).
- Generating initial codes and following the thread (MM produced descriptive statistics, followed a thread, and developed initial codes of the transcriptions using Nvivo 12 [42]).
- Searching for themes (MM reviewed each code and ordered them under headings, before discussing the ordered codes with JD to produce an initial triangulation matrix).
- Reviewing themes (EC, TM, and RS reviewed the themes, participant quotes, and matrix labels—resolving disagreements through discussion with MM and JD).
- Defining and naming themes (all authors discussed and agreed upon final themes).
- Producing the report.

### Ethical Approval

The trial was prospectively registered (ACTRN12617000681358) and approved by the Hunter New England Research Ethics Committee (Ref No. 11/03/16/4.05), University of Newcastle (Ref No. H-2011-0210), NSW Department of Education (SERAP 2011111), Maitland Newcastle Catholic School Diocese, Broken Bay Catholic School Diocese, Lismore Catholic School Diocese, Armidale Catholic School Diocese, and the Aboriginal Health and Medical Research Council.

### Availability of Data and Materials

The full data set supporting the conclusions of this article containing data not already included within the article or its additional files is available from the corresponding author on reasonable request.

## Results

The results are reported below by objective.

### Objective 1: Describe the Usage of the PA4E1 Program Website by In-School Champions and PE Teachers Using Quantitative Methods

There were a total of 273 users of the PA4E1 website during the whole study period. School users of the website were in-school champions (n=30) and PE teachers (n=198). Few principals (n=7) or school administration staff (n=2) registered for the program. Additionally, there were also nonschool users (n=20; ie, support officers and the PA4E1 research team) and unidentified users (n=16) of the program website.

Table 1 provides a breakdown of website usage for in-school champions and PE teachers from March 28, 2018 (term 3) to December 20, 2019 (term 9) (when the data were available by user) and for all program users from October 9, 2017, to December 20, 2019 (entire study period). In-school champions were the most frequent users of the program, with a mean of 48.0 sessions per user compared with 5.8 sessions per user by PE teachers. They also had higher total page views (276.6) and downloads (33.6) per user than PE teachers. PE teachers had fewer sessions (5.8), but these were of longer duration (10.5 vs 7.6 minutes) and included more page views on average (5.4 vs 3.4).

**Table 1:** Summary of Physical Activity 4 Everyone (PA4E1) website usage by user type.

Usage	In-school champions (n=30)^a^	Physical education teachers (n=198)^a^	All users (n=273)^b^
Sessions per user, mean	48.0	5.8	22.8
Page views per user, mean	276.6	57.0	179.4
Page views per session, mean	3.4	5.4	7.9
Session duration (minutes), mean	7.6	10.5	8.6
Downloads per user, mean	33.6	2.3	—

^a^Data were not available for the start of the program (October 9, 2017, to March 28, 2018). Data presented are from March 29, 2018, to December 20, 2019.

^b^The “All users” group includes in-school champions (n=30), PE teachers (n=198), principals (n=7), school administration staff (n=2) as well as nonschool users (n=20) and unidentified users (n=16).

The most frequently viewed pages by in-school champions were the home page (18.4% of all views), surveys and progress reports (15%), professional learning (10.3%), and resources (7.3%). PE teachers most frequently viewed professional learning (19.7% of all views) and the home page (9.6%).

A total of 90 different resources were downloaded from the website at least once by either an in-school champion or a PE teacher, equating to a total of 1007 downloads. The top 20 most downloaded resources accounted for more than half of all total downloads (n=559). Among the top downloaded resources were those directly assisting a particular implementation support strategy (Figure 1; see Table S1 in Multimedia Appendix 1) or physical activity practice (Figure 1; see Table S1 in Multimedia Appendix 1), which included a template for lesson observation (Practice 1), a student physical activity plan template (Practice 2), newsletter snippets (Practice 6), partnership agreement (Practice 7), and a school physical activity policy template (Practice 5).

**Figure 3:**
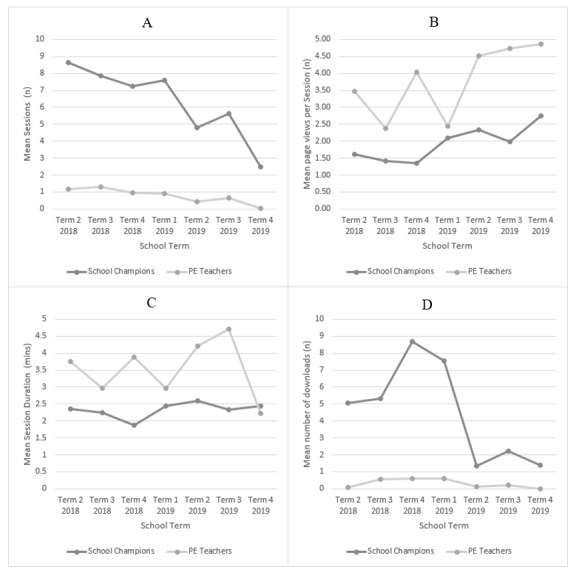
(A) Mean number of sessions, per term. (B) Mean number of page views per session, per term. (C) Mean session duration, per term. (D) Mean number of downloads, per term. Note that Term 4, 2017, and Term 1, 2018, data are not available (as described in the "Methods" section). PE: physical education.

The usage over time was explored by looking at the termly number of sessions, page views per session, average session duration, and number of downloads. Figure 3 shows these data per month for both in-school champions and PE teachers.

### Objective 2: Examine the Usage, Subjective Experience, and Usability of the PA4E1 Program Website From the Perspective of In-School Champions Using Mixed Methods

Of the 24 in-school champions invited to complete the think-aloud survey and usability test, 13 participated (54%). The average time to complete the survey and usability test was 20 minutes. In-school champions (9 females and 4 males) represented schools located in major cities (n=6), inner regional (n=6), and outer regional (n=1) regions [43]. Seven of the in-school champions also had responsibility for leading the health and PE programs in their school. All schools (9 government and 4 Catholic) were in the lower 50% of the Socio-Economic Indexes for Areas (SEIFA) of Relative Socioeconomic Disadvantage (suburb in lower 50% of NSW) [44]. School enrollments ranged from 348 to 1316 (mean 900 [SD 246]). Six schools had 10% or more Indigenous student enrollment [45]. Table 2 summarizes the meta-themes and themes for *delivery* and c*ontent*, respectively. Multimedia Appendix 4 includes the full triangulation matrix table and convergence labels.

**Table 2:** Summary of delivery and content meta-themes and themes.

Themes			Theme explained
**Delivery**			
	**Meta-theme**		The website was an acceptable and appropriate delivery mode. Usability of the website was high.
		Theme 1	The website complemented other delivery modes, but in-school champions preferred the other delivery modes (support officer and face-to-face).
		Theme 2	The website was used as a “utility” delivery mode (ie, used only when required, not as the first delivery mode of choice).
		Theme 3	If the program were delivered statewide, the website would be useful, though embedding within other systems that teachers already used may be helpful.
		Theme 4	Mixed reactions toward the possible addition of a website chat feature with a support officer available to video call during business hours.
		Theme 5	The discussion forum was not used, but in-school champions reported that it could be potentially useful, perhaps if delivered differently.
**Content**			
	**Meta-theme**		The website content was generally acceptable and appropriate, with a few specific suggestions for improvement.
		Theme 1	A lack of notifications (or prompts) to highlight new things in the website reduced return traffic to the website.
		Theme 2	The lesson observation form (a resource for Practice 1) was difficult to find on the website, it was difficult to track lesson observation completion, and some usability issues with completing out of internet range.
		Theme 3	Termly surveys (Support Strategy 7) were generally acceptable and completed.
		Theme 4	The resources were acceptable and downloaded frequently.

### Delivery

#### Meta-Theme: The Website Was an Acceptable and Appropriate Delivery Mode. Usability of the Website Was High (Triangulation Convergence Label: Agree)

Quantitative data indicated in-school champions agreed or strongly agreed that the PA4E1 website was acceptable and appropriate. For in-school champions (n=13), the mean acceptability of the intervention measure was 4.52 (SD 0.04) out of 5, and the mean Intervention Appropriateness Measure was 4.46 (SD 0.0) out of 5, both indicating agreement that the website is acceptable and appropriate. Additionally, the mean in-school champions’ (n=13) overall User Engagement Scale score was 3.4 (SD 0.7) out of 5 [32]. The mean individual dimension scores from the User Engagement Scale were 4.2 (SD 0.1), 3.7 (SD 0.5), 3.4 (SD 0.3), and 2.4 (SD 0.3) for aesthetic appeal, reward factor, perceived usability, and focused attention, respectively.

In-school champions found the usability of the website to be “good.” The overall in-school champion (n=13) Systems Usability Scale score was 77.7, which corresponds to a “good” website within the “acceptable” range of the Systems Usability Scale [31,37]. Additionally, the majority of the 3 navigation tasks were completed successfully by in-school champions (74% [29/39] of all tasks completed successfully). The average time to task completion was 71 seconds per task. The discussion forum and community links tasks had higher success rates (11/13 [85%] and 12/13 [92%], respectively) than the lesson observation task (6/13, 46%). Time to complete each task was 91, 66, and 55 seconds for discussion forum, community links, and lesson observation tasks, respectively. No tasks were abandoned.

Qualitative data also indicated good acceptability, appropriateness, and usability of the website delivery mode. Some example quotes are as follows:

...it's set out very clearly, easy to use, easy to access, and is updated relatively frequently.

So, we all know that teachers are the time poor people of the world. So definitely having an online mode is very, very suitable.

#### Theme 1: The Website Complemented Other Delivery Modes, But In-School Champions Preferred the Other Delivery Modes (Support Officer and Face-to-Face; Triangulation Convergence Label: Agree)

Quantitative data showed schools utilized the website (Figure 1). In-school champions also relied on the other delivery modes, for example, contact logs between support officers and in-school champions indicated that 18/24 schools received a face-to-face contact with their support officer at least once a term in the first 12 months. In the second 12 months, this dropped to 8/24 schools, though the criteria for this implementation support strategy were different for the second 12 months (Multimedia Appendix 1). However, weekly emails or phone calls between support officers and in-school champions occurred in 16/24 schools during the first 12 months, but this increased to 22/24 schools in the second 12 months. Additionally, the 2 face-to-face workshops were well attended; 23/24 in-school champions attended the first workshop at the start of the program while 22/24 attended the second workshop at the midpoint of the program.

Qualitative data indicated that in-school champions used the website to complement interactions with their support officer. The website often was not the first point of call, and it may not be able to fully replace face-to-face and direct contact methods from the support officers. The website may be particularly useful for after-hours support (eg, on weekends and evenings). Example quotes:

You've obviously got your Support Officer as well [as the website], but it's good just to have this, this portal [website].

Yes [I like the website], but it needs to be accompanied by other things like face-to-face as well.

...it is an invaluable experience to meet, at a common place, where you can actually roll out, the initial delivery, and training, for the first time that people are running PA4E1. However, as said before, online delivery should remain part of this program.

...teachers are often not being able to get this extra, sort of, work, or professional learning done during their actual hours of being at school. So I often access the website from home, after hours, on the weekend.

#### Theme 2: The Website Was Used as a “Utility” Delivery Mode (ie, Used Only When Required, Not as the First Delivery Mode of Choice; Triangulation Convergence Label: Agree)

Quantitative data from objective 1 indicated that in-school champions used the website on average every fortnight, corresponding to 6.3 sessions per term (SD 2.0). Qualitative data identified that the website was perhaps not used to its full potential, but instead it was used for its core functions (eg, termly surveys, professional learning), rather than to peruse additional ideas and resources. Example quotes:

To be honest I don't think I've used PA4E1 online [the website], probably to its full potential.

Yeah well obviously I haven't really used this [pointing cursor at the resources section] as much as I should've. I have used some of these [resources] though...

I'm aware of most kind of, components of it [the website], but there are things I'm not [aware of]...

#### Theme 3: If the Program Were Delivered Statewide, the Website Would Be Useful, Though Embedding Within Other Systems That Teachers Already Used May Be Helpful (Triangulation Convergence Label: Slightly Agree)

All in-school champions (13/13) agreed that online delivery of the PA4E1 program should remain. The majority of in-school champions (12/13) suggested the PA4E1 website would be a suitable mode of delivery in its current form should the PA4E1 program be rolled out to schools statewide.

There were conflicting suggestions emerging from the qualitative data. In-school champions indicated that while a website would be useful for statewide delivery of the program, it could become another platform that teachers are asked to use, so it would be better embedding any roll out within existing platforms. Example quotes:

...it is another platform that we need to access in order to implement our teaching.

I do think it is quite suitable, to roll out in its current form if there was to be no changes.

The more people that are on it [the website] as well, I think the more discussion that would kind of, be generated....

#### Theme 4: Mixed Reactions Toward the Possible Addition of a Website Chat Feature With a Support Officer Available to Video Call During Business Hours (Triangulation Convergence Label: Slightly Agree)

Quantitative data were generally supportive; the majority of in-school champions (11/13) liked the idea that support officers would be available during working hours for support within the PA4E1 website via video, audio, or text chat features. Qualitative data were mixed, with some supporting the feature, while some preferring traditional contact methods such as phone call and face-to-face. Some example quotes:

Supportive: it's [a] great [idea], because whenever you can squeeze it in in your free period there's someone there to talk to you about so you don't have to wait until the [in-School] Champion or someone in the know is available to help you…that's fantastic.

Neutral: I mean it might ease the load on the Support Officers having to kind of travel everywhere [around the NSW state], 'cause then you have that option to sit and talk to them like you would face-to-face. But yeah I mean I like it, but I don't dislike it, but I don't know if it's hugely necessary.

Nonsupportive: I dislike because I would prefer face-to-face. Email works just as well, and a phone call if necessary.

#### Theme 5: The Discussion Forum Wasn’t Used, But In-School Champions Reported It Could Be Potentially Useful, Perhaps If Delivered Differently (Triangulation Convergence Label: Agree)

Quantitative usage data showed that use of the discussion forum was very low. There were 3 discussion forum posts made by support officers to generate content; however, there were 0 discussion forum posts by in-school champions or PE teachers during the entire program. In response, the support officers created a Facebook group for in-school champions which was established on November 7, 2018. From the launch of the Facebook group until the end of the program, there were 10 posts by the PA4E1 team (support officers and staff) and 12 posts by in-school champions in the Facebook group.

Qualitative data suggested the discussion forum was not very useful, and may be better delivered within the website, or could be better delivered using Facebook. Example quotes:

Yep. It's [the discussion forum]...a little bit limited.

I think this was mentioned at the training day, the suggestion that a discussion forum could pop up on the front page...so, it was the, kind of, first thing you saw, and the first thing that popped up, whenever you went there.

I don't think that the discussion forum is a good mode of communication. It appears that no one's really using it. Perhaps a different form of social media such as Facebook, which is happening, that more people use.

### Content

#### Meta-Theme: The Website Content Was Generally Acceptable and Appropriate, With a Few Specific Suggestions for Improvement (Triangulation Convergence Label: Agree)

As per quantitative data from the previous meta-theme relating to *delivery*, the majority of in-school champions agreed or strongly agreed that the website was acceptable, appropriate, and had good usability.

Qualitative data also indicated the website content was appropriate. Example quotes:

Yeah, I think the online portal in its current form is kind of suitable, I think a few minor changes and adjustments would make it better.

...the online portal is essential with implementation of the program. You couldn't do it without it. It's your go-to resource.

#### Theme 1: A Lack of Notifications (or Prompts) to Highlight New Things in the Website Reduced Return Traffic to the Website (Triangulation Convergence Label: Silence)

No quantitative data were available for this theme. Qualitative data highlighted that notifications would have been useful to highlight updates to the website, which may subsequently increase usage.

...sometimes new things just pop up that I didn't really realize were there

Maybe also having some form of like, notification so in the top right hand corner...so that you know, like, you can see new...things that are kind of happening...

...the portal [website] is not something I log onto everyday. As we all kind of said, like, everyone goes onto Facebook and things like that. So, that's where- and you get notifications that pops up on your phone, where often I don't know that things have been posted in the discussion forum, until I come back on, and often that's quite a while after they were posted.

#### Theme 2: The Lesson Observation Form (a Resource for Practice 1) Was Difficult to Find on the Website, It Was Difficult to Track Lesson Observation Completion, and Some Usability Issues With Completing Out Of Internet Range (Triangulation Convergence Label: Agree)

More than half of in-school champions (7/13) were unable to find the lesson observation section on the website. Qualitative data also indicated that in-school champions had difficulty finding the lesson observations, tracking PE teachers’ completion of these observations, and also some issues with signal to the website while out of the internet range. Example quotes:

You do have to scroll down a little bit on the page [to find the Lesson Observation Form], and it is like, quite a small link, which I know some staff at our school have had trouble, kind of, finding it.

...you just kind of have to do a bit of a tally of how many observations one teacher has had.

Sometimes when we were out of mobile range or if this person didn't have a mobile, it didn't quite, ah, work out.

#### Theme 3: Termly Surveys (Support Strategy 7) Were Generally Acceptable and Completed (Triangulation Convergence Label: Slightly Agree)

From 0 to 12 months, 24/24 in-school champions completed all termly surveys and received the feedback reports from these surveys. From 12 to 24 months, 21/24 in-school champions completed all termly surveys and received feedback reports (3 schools missed 1 survey each). Few principals received the feedback reports, 7/24 principals had registered accounts with the program website and therefore received the feedback reports via email. The remaining principals did not directly receive the feedback reports, but may have been shown to them by their in-school champions. Qualitative data were scarce, but some support for the acceptability of the termly surveys was provided. Example quote:

...the termly surveys, I mean this was handy to see where we were at but I suppose before I did the survey I kind of had an idea of what we did and didn't do well.

#### Theme 4: The Resources Were Acceptable and Downloaded Frequently (Triangulation Convergence Label: Agree)

A total of 90 different resources were downloaded from the website at least once by either an in-school champion or a PE teacher, equating to a total of 1007 downloads. Example quotes:

I really love this particular resource section. Yeah, look, I just can't say enough about, you know, what I love about it.

I loved the graphics and the images. I've really, I really liked working with the Physical Activity Policy template. I thought it looked really professional, yeah, I- I did really like...those aspects.

## Discussion

### Principal Findings

We have previously reported that the multimode implementation support strategies used in the PA4E1 scale-up trial increased the implementation of physical activity–promoting practices in lower socioeconomic secondary schools in New South Wales, Australia [32]. The results presented in this paper expand on these findings with quantitative and mixed methods process evaluation data on the role of the PA4E1 website in delivery of the implementation support strategies of the PA4E1 program. To our knowledge, this is the first comprehensive process evaluation of a school-based physical activity program to focus exclusively on the digital delivery mode of a multidelivery mode implementation support strategy. The primary findings indicate that usage of the website by both the in-school champions and PE teachers was high, which aligns with the high fidelity and reach of the implementation support found at 12 months reported elsewhere [32]. As intended, in-school champions had more frequent website use than PE teachers. The results of the mixed methods analysis indicated that both the delivery and content of the website were acceptable and appropriate. A number of usability issues were identified and are included as suggested modifications for future iterations of the website.

The findings support the value of the website within a multidelivery mode implementation intervention to support schools to implement physical activity promoting practices. Although other digital health interventions targeting physical activity have often suffered with issues of engagement [6,10,46], the website delivering implementation support for the PA4E1 program does not appear to have impaired the potential of the program to have a positive impact. As discussed by Sebire et al [47], embracing technology in school physical activity interventions may be an effective way to efficiently deliver content, for example, by reducing challenges related to limited time for training.

Completing this study about website engagement as a component of our broader process evaluation has provided information not typically collected within trials of school-based physical activity programs. Previous studies have either omitted evaluation of the website delivery mode [48,49], or largely relied on quantitative website usage data to assess usage engagement [9,50-52]. These studies have reported varying levels of website usage, with studies’ usage engagement varying between pilot and full trial [52,53]. Focusing only on usage data ignores the other component of engagement, subjective experience [9]. This study includes both quantitative and qualitative data exploring both usage and subjective experience engagement [9], with both data sets being triangulated to report high acceptability and appropriateness of the delivery and content of the website. Usage data showed that in-school champions and PE teachers accessed the website frequently, though this appeared to decline over time (Figure 3). As intended by the design of the PA4E1 implementation support strategies (Multimedia Appendix 1), in-school champions accessed the website more frequently than PE teachers. Additionally, compared with other websites, the PA4E1 website has a rating of “good” usability [37,40].

The study was novel in its use of “think-aloud” methodology to explore teachers’ experiences of using a website to support the delivery of a health-based intervention. Think-aloud methodologies have largely been used to inform the development of websites and apps for use directly with the target user of the health intervention [54-58] (eg, an app to support weight management among adults with diabetes [54]). By contrast, we used a think-aloud methodology with teachers (in-school champions) who have been accessing a website to support the delivery of the PA4E1 program to adolescent students. Previous studies using the think-aloud methodology [54-58] have found it to be useful for identifying usability and subjective experience issues. However, the procedure used in these studies involved the presence of a researcher, which may have affected participants’ reactions. Our think-aloud procedure allowed in-school champions to respond to the think-aloud survey and usability test remotely, using their own devices, in their own time. Such a method is likely to be more ecologically valid than those involving the presence of researchers, at research sites, using research devices [59].

### Intervention Refinements

This process evaluation revealed several refinements and suggestions for the PA4E1 website. In-school champions suggested the website was used in conjunction with other delivery modes (face-to-face, phone, email) and that these delivery modes of the implementation support strategies were also highly valued, which was additionally supported by usage data of multiple delivery modes. In addition, while the program website was acceptable and appropriate in its current format, it was often not the “first-choice” for in-school champions; often, in-school champions would use other delivery modes first. In-school champions also suggested the website could be embedded within other systems that teachers already access, such as state education portals. Therefore, we suggest future iterations of the PA4E1 program should carefully consider the balance of delivery modes used to deliver the implementation support strategies.

In-school champions suggested that both a website chat feature (to access support officers) and a discussion forum (to chat with other in-school champions) may be useful, although this was not a unanimous suggestion. Strategies to increase engagement with such features may be required, which was further highlighted by in-school champions who suggested the addition of notifications within the website and via email to promote usage of the website and highlight new content. Other features that may improve engagement include social networking platforms [60], social support, and behavioral prompts [61]. Finally, the lesson observation form (see Practice 1 outlined in Multimedia Appendix 1, Table S2) was difficult to find on the website (task completion = 46% [6/13]) and in-school champions reported that this could be made more prominent within the website.

Future iterations of a website supporting PA4E1 delivery may benefit from user testing and refinement prior to rolling out the website for delivery [62]. Such formative evaluations are common to inform e-commerce websites and from the human–computer interaction literature [62].

### Strengths and Limitations

A key strength of this study is the detailed mixed methods design, triangulating multiple data sources to provide a more coherent and actionable set of themes from the data. Another strength is the use of an ecologically valid remote think-aloud survey and usability test procedure, to collect in-school champions experiences of using the PA4E1 website.

Limitations of this study include the low response rate to the think-aloud survey and usability test. Despite the remote nature of the survey, some respondents found it difficult to use the Loop 11 think-aloud survey and usability test software. Just over half of in-school champions (13/24, 54%) responded to the think-aloud survey and usability test. This may have introduced response bias, whereby those who responded were more likely to report high acceptability and appropriateness of the website. However, with regard to sample size, 5 participants are deemed sufficient for detecting most usability problems [63]. Another limitation was the inability to segregate user types prior to term 2, 2018. This was due to a technical difficulty in collecting the data in Google Analytics that could only be fixed prospectively from school term 2, 2018.

### Conclusion

The results of this study provide context to support the primary outcome of the trial, highlighting that both in-school champions and PE teachers used the program website, and acceptability and appropriateness of the website were high among in-school champions. Our study has focused on the website delivery mode of a multidelivery mode implementation support strategy to support schools to implement physical activity promoting practices. We will publish further process evaluation results elsewhere, focusing on the remaining components of our process evaluation protocol [28].

## Appendix

Multimedia Appendix 1 Supplementary file 1. Supplementary Table 1 and Supplementary Table 2.

Multimedia Appendix 2 Supplementary file 2. AMUsED framework.

Multimedia Appendix 3 Supplementary file 3. Think-aloud survey and usability test - questions and tasks.

Multimedia Appendix 4 Supplementary file 4. Triangulation matrix.

## Abbreviations

AMUsED: Analyzing and Measuring Usage and Engagement Data
PA4E1: Physical Activity 4 Everyone
PE: physical education
SEIFA: Socio-Economic Indexes for Areas

## References

[1] Guthold R, Stevens G, Riley L, Bull F (2018). Worldwide trends in insufficient physical activity from 2001 to 2016: a pooled analysis of 358 population-based surveys with 1·9 million participants. Lancet Glob Health.

[2] Guthold R, Stevens GA, Riley LM, Bull FC (2020). Global trends in insufficient physical activity among adolescents: a pooled analysis of 298 population-based surveys with 1·6 million participants. Lancet Child Adolesc Health.

[3] (2018). Global Action Plan on Physical Activity 2018-2030: More Active People for a Healthier World.

[4] McCrabb S, Lane C, Hall A, Milat A, Bauman A, Sutherland R, Yoong S, Wolfenden L (2019). Scaling-up evidence-based obesity interventions: A systematic review assessing intervention adaptations and effectiveness and quantifying the scale-up penalty. Obes Rev.

[5] Katz D (2009). School-based interventions for health promotion and weight control: not just waiting on the world to change. Annu Rev Public Health.

[6] Davies CA, Spence JC, Vandelanotte C, Caperchione CM, Mummery W (2012). Meta-analysis of internet-delivered interventions to increase physical activity levels. Int J Behav Nutr Phys Act.

[7] Romeo A, Edney S, Plotnikoff R, Curtis R, Ryan J, Sanders I, Crozier A, Maher C (2019). Can Smartphone Apps Increase Physical Activity? Systematic Review and Meta-Analysis. J Med Internet Res.

[8] Bort-Roig J, Gilson ND, Puig-Ribera A, Contreras RS, Trost SG (2014). Measuring and influencing physical activity with smartphone technology: a systematic review. Sports Med.

[9] Perski O, Blandford A, West R, Michie S (2017). Conceptualising engagement with digital behaviour change interventions: a systematic review using principles from critical interpretive synthesis. Transl Behav Med.

[10] Mclaughlin M, Delaney T, Hall A, Byaruhanga J, Mackie P, Grady A, Reilly K, Campbell E, Sutherland R, Wiggers J, Wolfenden L (2021). Associations Between Digital Health Intervention Engagement, Physical Activity, and Sedentary Behavior: Systematic Review and Meta-analysis. J Med Internet Res.

[11] Maia CLB, Furtado ES, Marcus A (2016). A systematic review about user experience evaluation. Design, User Experience, and Usability: Design Thinking and Methods. DUXU 2016. Lecture Notes in Computer Science, vol 9746.

[12] Moore GF, Audrey S, Barker M, Bond L, Bonell C, Hardeman W, Moore L, O'Cathain A, Tinati T, Wight D, Baird J (2015). Process evaluation of complex interventions: Medical Research Council guidance. BMJ.

[13] Love R, Adams J, van Sluijs Esther M F (2019). Are school-based physical activity interventions effective and equitable? A meta-analysis of cluster randomized controlled trials with accelerometer-assessed activity. Obes Rev.

[14] Jong ST, Croxson CH, Guell C, Lawlor ER, Foubister C, Brown HE, Wells EK, Wilkinson P, Vignoles A, van Sluijs EM, Corder K (2020). Adolescents' perspectives on a school-based physical activity intervention: A mixed method study. J Sport Health Sci.

[15] Cosme Chavez R, Nam EW (2020). Process Evaluation of a School-Based Program Aimed at Preventing Obesity in Adolescents from Lima and Callao, Peru. Int J Environ Res Public Health.

[16] Sebire SJ, Banfield K, Jago R, Edwards MJ, Campbell R, Kipping R, Blair PS, Kadir B, Garfield K, Matthews J, Lyons RA, Hollingworth W (2019). A process evaluation of the PLAN-A intervention (Peer-Led physical Activity iNtervention for Adolescent girls). BMC Public Health.

[17] Gorely T, Harrington DM, Bodicoat DH, Davies MJ, Khunti K, Sherar LB, Tudor-Edwards R, Yates T, Edwardson CL (2019). Process evaluation of the school-based Girls Active programme. BMC Public Health.

[18] Proctor E, Silmere H, Raghavan R, Hovmand P, Aarons G, Bunger A, Griffey R, Hensley M (2011). Outcomes for implementation research: conceptual distinctions, measurement challenges, and research agenda. Adm Policy Ment Health.

[19] Gebremariam MK, Arah OA, Bergh IH, Andersen LF, Bjelland M, Grydeland M, Lien N (2019). Factors affecting the dose of intervention received and the participant satisfaction in a school-based obesity prevention intervention. Prev Med Rep.

[20] Parrish A, Trost SG, Howard SJ, Batterham M, Cliff D, Salmon J, Okely AD (2018). Evaluation of an intervention to reduce adolescent sitting time during the school day: The 'Stand Up for Health' randomised controlled trial. J Sci Med Sport.

[21] Bonde AH, Stjernqvist NW, Sabinsky MS, Maindal HT (2018). Process evaluation of implementation fidelity in a Danish health-promoting school intervention. BMC Public Health.

[22] Bellew B, Nau T, Smith B, Bauman A (2020). Getting Australia Active III: A systems approach to physical activity for policy makers.

[23] (2021). What is a Health Promoting School?. World Health Organization.

[24] Milton Karen, Cavill Nick, Chalkley Anna, Foster Charlie, Gomersall Sjaan, Hagstromer Maria, Kelly Paul, Kolbe-Alexander Tracy, Mair Jacqueline, McLaughlin Matthew, Nobles James, Reece Lindsey, Shilton Trevor, Smith Ben J, Schipperijn Jasper (2021). Eight Investments That Work for Physical Activity. J Phys Act Health.

[25] Sutherland R, Campbell E, Lubans DR, Morgan PJ, Okely AD, Nathan N, Wolfenden L, Jones J, Davies L, Gillham K, Wiggers J (2013). A cluster randomised trial of a school-based intervention to prevent decline in adolescent physical activity levels: study protocol for the ‘Physical Activity 4 Everyone’ trial. BMC Public Health.

[26] Sutherland R, Campbell E, Lubans DR, Morgan PJ, Okely AD, Nathan N, Wolfenden L, Wiese J, Gillham K, Hollis J, Wiggers J (2016). 'Physical Activity 4 Everyone' school-based intervention to prevent decline in adolescent physical activity levels: 12 month (mid-intervention) report on a cluster randomised trial. Br J Sports Med.

[27] Sutherland RL, Campbell EM, Lubans DR, Morgan PJ, Nathan NK, Wolfenden L, Okely AD, Gillham KE, Hollis JL, Oldmeadow CJ, Williams AJ, Davies LJ, Wiese JS, Bisquera A, Wiggers JH (2016). The Physical Activity 4 Everyone Cluster Randomized Trial: 2-Year Outcomes of a School Physical Activity Intervention Among Adolescents. Am J Prev Med.

[28] Mclaughlin M, Duff J, Sutherland R, Campbell E, Wolfenden L, Wiggers J (2020). Protocol for a mixed methods process evaluation of a hybrid implementation-effectiveness trial of a scaled-up whole-school physical activity program for adolescents: Physical Activity 4 Everyone (PA4E1). Trials.

[29] Sutherland R, Campbell E, Nathan N, Wolfenden L, Lubans DR, Morgan PJ, Gillham K, Oldmeadow C, Searles A, Reeves P, Williams M, Evans N, Bailey A, Morrison R, McLaughlin M, Wiggers J (2019). A cluster randomised trial of an intervention to increase the implementation of physical activity practices in secondary schools: study protocol for scaling up the Physical Activity 4 Everyone (PA4E1) program. BMC Public Health.

[30] Sutherland R, Reeves P, Campbell E, Lubans DR, Morgan PJ, Nathan N, Wolfenden L, Okely AD, Gillham K, Davies L, Wiggers J (2016). Cost effectiveness of a multi-component school-based physical activity intervention targeting adolescents: the 'Physical Activity 4 Everyone' cluster randomized trial. Int J Behav Nutr Phys Act.

[31] Hollis JL, Sutherland R, Campbell L, Morgan PJ, Lubans DR, Nathan N, Wolfenden L, Okely AD, Davies L, Williams A, Cohen KE, Oldmeadow C, Gillham K, Wiggers J (2016). Effects of a 'school-based' physical activity intervention on adiposity in adolescents from economically disadvantaged communities: secondary outcomes of the 'Physical Activity 4 Everyone' RCT. Int J Obes (Lond).

[32] Sutherland R, Campbell E, McLaughlin M, Nathan N, Wolfenden L, Lubans DR, Morgan PJ, Gillham K, Oldmeadow C, Searles A, Reeves P, Williams M, Kajons N, Bailey A, Boyer J, Lecathelinais C, Davies L, McKenzie T, Hollis J, Wiggers J (2020). Scale-up of the Physical Activity 4 Everyone (PA4E1) intervention in secondary schools: 12-month implementation outcomes from a cluster randomized controlled trial. Int J Behav Nutr Phys Act.

[33] Miller S, Ainsworth B, Yardley L, Milton A, Weal M, Smith P, Morrison L (2019). A Framework for Analyzing and Measuring Usage and Engagement Data (AMUsED) in Digital Interventions: Viewpoint. J Med Internet Res.

[34] SAS (2014). Enterprise Guide 6. SAS.

[35] Charters E (2003). The Use of Think-aloud Methods in Qualitative Research An Introduction to Think-aloud Methods. Brock Education Journal.

[36] Loop11.

[37] Brooke J (1996). SUS: A quick and dirty usability scale. Systems Usability Scale.

[38] O’Brien HL, Cairns P, Hall M (2018). A practical approach to measuring user engagement with the refined user engagement scale (UES) and new UES short form. International Journal of Human-Computer Studies.

[39] Weiner BJ, Lewis CC, Stanick C, Powell BJ, Dorsey CN, Clary AS, Boynton MH, Halko H (2017). Psychometric assessment of three newly developed implementation outcome measures. Implementation Sci.

[40] Bangor A, Kortum P, Miller J (2009). Determining what individual SUS scores mean: adding an adjective rating scale. J Usability Studies.

[41] Braun V, Clarke V (2006). Using thematic analysis in psychology. Qualitative Research in Psychology.

[42] (2018). NVivo.

[43] Australian Bureau of Statistics (2011). Statistical Geography Volume 1- Australian Standard Geogrphical Classification (ASGC).

[44] Australian Bureau of Statistics (2011). 2033. 0.55.001 001 - Socio-economic Indexes for Areas (SEIFA) - State Suburb (SSC) Index of Relative Socio-economic Disadvantage.

[45] (2019). My School is a resource for parents, educators and the community to find information about each of Australia's schools. Australian Curriculum, Assessment and Reporting Authority.

[46] Vandelanotte C, Müller Andre M, Short CE, Hingle M, Nathan N, Williams SL, Lopez ML, Parekh S, Maher CA (2016). Past, Present, and Future of eHealth and mHealth Research to Improve Physical Activity and Dietary Behaviors. J Nutr Educ Behav.

[47] Sebire SJ, Kesten JM, Edwards MJ, May T, Banfield K, Tomkinson K, Blair PS, Bird EL, Powell JE, Jago R (2016). Using self-determination theory to promote adolescent girls' physical activity: Exploring the theoretical fidelity of the Bristol Girls Dance Project. Psychol Sport Exerc.

[48] Smedegaard S, Brondeel R, Christiansen LB, Skovgaard T (2017). What happened in the 'Move for Well-being in School': a process evaluation of a cluster randomized physical activity intervention using the RE-AIM framework. Int J Behav Nutr Phys Act.

[49] Kostamo K, Jallinoja P, Vesala KM, Araújo-Soares Vera, Sniehotta FF, Hankonen N (2019). Using the critical incident technique for qualitative process evaluation of interventions: The example of the "Let's Move It" trial. Soc Sci Med.

[50] Lubans DR, Smith JJ, Skinner G, Morgan PJ (2014). Development and implementation of a smartphone application to promote physical activity and reduce screen-time in adolescent boys. Front Public Health.

[51] Jong ST, Croxson CHD, Foubister C, Brown HE, Guell C, Lawlor ER, Wells EK, Wilkinson PO, Wilson ECF, van Sluijs EMF, Corder K (2020). Reach, Recruitment, Dose, and Intervention Fidelity of the GoActive School-Based Physical Activity Intervention in the UK: A Mixed-Methods Process Evaluation. Children.

[52] Corder K, Sharp SJ, Jong ST, Foubister C, Brown HE, Wells EK, Armitage SM, Croxson CHD, Vignoles A, Wilkinson PO, Wilson ECF, van Sluijs EMF (2020). Effectiveness and cost-effectiveness of the GoActive intervention to increase physical activity among UK adolescents: A cluster randomised controlled trial. PLoS Med.

[53] Corder K, Brown HE, Schiff A, van Sluijs EMF (2016). Feasibility study and pilot cluster-randomised controlled trial of the GoActive intervention aiming to promote physical activity among adolescents: outcomes and lessons learnt. BMJ Open.

[54] Simpson SA, Matthews L, Pugmire J, McConnachie A, McIntosh E, Coulman E, Hughes K, Kelson M, Morgan-Trimmer S, Murphy S, Utkina-Macaskill O, Moore L (2020). An app-, web- and social support-based weight loss intervention for adults with obesity: the HelpMeDoIt! feasibility RCT. Public Health Res.

[55] Nurmi J, Knittle K, Ginchev T, Khattak F, Helf C, Zwickl P, Castellano-Tejedor C, Lusilla-Palacios P, Costa-Requena J, Ravaja N, Haukkala A (2020). Engaging Users in the Behavior Change Process With Digitalized Motivational Interviewing and Gamification: Development and Feasibility Testing of the Precious App. JMIR Mhealth Uhealth.

[56] Castle EM, Greenwood J, Chilcot J, Greenwood SA (2021). Usability and experience testing to refine an online intervention to prevent weight gain in new kidney transplant recipients. Br J Health Psychol.

[57] Simons D, De Bourdeaudhuij I, Clarys P, De Cocker K, Vandelanotte C, Deforche B (2018). A Smartphone App to Promote an Active Lifestyle in Lower-Educated Working Young Adults: Development, Usability, Acceptability, and Feasibility Study. JMIR Mhealth Uhealth.

[58] Poppe L, Van der Mispel C, De Bourdeaudhuij I, Verloigne M, Shadid S, Crombez G (2017). Users' thoughts and opinions about a self-regulation-based eHealth intervention targeting physical activity and the intake of fruit and vegetables: A qualitative study. PLoS One.

[59] Shiffman S, Stone AA, Hufford MR (2008). Ecological momentary assessment. Annu Rev Clin Psychol.

[60] Petersen JM, Prichard I, Kemps E (2019). A Comparison of Physical Activity Mobile Apps With and Without Existing Web-Based Social Networking Platforms: Systematic Review. J Med Internet Res.

[61] Bardus M, van Beurden SB, Smith JR, Abraham C (2016). A review and content analysis of engagement, functionality, aesthetics, information quality, and change techniques in the most popular commercial apps for weight management. Int J Behav Nutr Phys Act.

[62] Blandford A, Gibbs J, Newhouse N, Perski O, Singh A, Murray E (2018). Seven lessons for interdisciplinary research on interactive digital health interventions. Digit Health.

[63] Faulkner L (2003). Beyond the five-user assumption: benefits of increased sample sizes in usability testing. Behav Res Methods Instrum Comput.

