# Supplementary material for: Evaluating Digital Program Support for the Physical Activity 4 Everyone (PA4E1) School Program: Mixed Methods Study
Source: JMIR Pediatr Parent. 2021 Jul 26;4(3):e26690. doi: 10.2196/26690 (PMC8367175; doi:10.2196/26690)
Supplement: Multimedia Appendix 1 [file pediatrics_v4i3e26690_app1.docx]

| \| **Supplementary File 1** \| \| --- \|   **Supplementary File 1, Table 1:** Role of the program website in the delivery of the implementation support strategies | |
| --- | --- | --- |
| **PA4E1 Implementation Support Strategies (n=7) and sub-strategies (n=23)** | **Role of the website delivery mode^** |
| **1. Executive and leadership support** |  |
| **1.1:** PA4E1 Partnership agreement signed by school executive. | Draft template agreement stored in Resources tab. |
| **1.2:** New or existing school committee formed to oversee program. | None |
| **1.3:** The School committee is inclusive of in-School Champion and school executive to oversee the program. | None |
| **1.4:** Committee met at least once per term. | None |
| **2. Embedded school staff: in-School Champion** |  |
| **2.1:** An existing school PE teacher is allocated the role of in-School Champion to support implementation for full 24 months^#^. | In-School Champions were provided access to the program website at the start of the program (school Term 4 2017). |
| **2.2:** The position was funded by the NSW Department of Health, half day per week (equivalent to $350AUD a fortnight). | None |
| **3. External implementation support** |  |
| **3.1:** Health Promotion Support Officer (ideally a trained PE teacher) appointed to support schools with the program. | Support Officers were provided access to the program website and could view their schools progress. |
| **3.2:** Health Promotion Support Officer was co-located within the relevant local health district. | None |
| **3.3:** In the first 12 months, weekly contact was made by Support Officer with in-School Champion via phone, email and/or face-to-face site visits. In the second 12 months^#^, contact was made with in-School Champion via phone, email and/or face-to-face site visits according to the following high dose/low dose protocol matched to school practice uptake in the preceding term (e.g. Term 5 informs Term 6).   1. Schools adopting four or less practices received 2 face-to-face contact points, as well as 6 emails or phonecalls in the term. 2. Schools adopting five or more practices 1 face-to-face contact point and seven emails or phone calls in the term. | None |
| **3.4:** In the first 12 months, Support Officer and in-School Champion have a face-to-face contact at least once a term. In the second 12 months^#^, Support Officers and in-School Champions should have had face-to-face site visits according to the following high dose/low dose protocol matched to school physical activity practice uptake in the preceding term (e.g., Term 5 informs Term 6).   1. Schools adopting four or less practices receive 2 face-to-face contacts 2. Schools adopting five or more practices receive 1 face-to-face contact | None |
| **4. Teacher professional learning** |  |
| **4.1:** In-School Champion training –1-day of face-to-face training session was hosted by PA4E1 implementation in the first 12 months (Term 4 2017) and the second 12 months^#^ (Term 4 2018). Accommodation, meals and transport costs were covered by the NSW Department of Health. | The Resources tab of the website hosted workshop materials and an audio recording of the workshop. |
| **4.2:** Quality PE training for all PE teachers - 6 x 10-minute online training videos followed by knowledge check short quizzes focused on the SAAFE principles were delivered via a password protected program website. | The Professional Learning tab housed the 6 professional learning modules as well as two summary modules. Each module included a 10 minute video and a knowledge check quiz after each video, and tracked PE teacher completion. |
| **4.3*:** Enhanced school sport training – in-School Champions and other teachers involved in delivering the program could attend an existing 1 day face-to-face Resistance Training for Teens workshop offered by the NSW Department of Education (School Sport Unit), or equivalent training run by PA4E1 implementation team (not accredited). Course costs to be paid by project for in-School Champion, but not for other teachers. | The Resources tab housed resources relating to the enhanced school sport program. |
| **4.4*:** School physical activity policy training – in-School Champion offered existing online training run by the NSW Department of Education School Sport Unit (Government schools only, n=19) (59). | The Resources tab housed resources relating to the school physical activity policy. |
| **5. Resources** |  |
| **5.1*:** Printed posters outlining Quality PE principles (SAAFE Principles (60)) to be displayed in PE department delivered to in-School Champions. | None |
| **5.2*:** A $100AUD physical activity equipment voucher was provided to support the delivery of recess and lunchtime physical activity. | None |
| **5.3*:** Equipment provided to support the delivery of recess and lunchtime physical activity enhanced schools sport program (5 Gymsticks/school) | None |
| **5.4:** Electronic resources housed on the program website (PA4E1 online) included:   - - overview of program presentation (Microsoft PowerPoint presentation)   - project milestones to be achieved each term (over 4 terms)   - online quality PE training (SAAFE Principle videos (6 videos - one overview and one per Principle) and worksheet, peer observation materials)   - student personal physical activity plan templates   - recess and lunch resources   - policy templates   - examples of community physical activity providers   tips and frequently asked questions | The website housed all the resources on the Resources tab. |
| **6. Provision of prompts and reminders** |  |
| **6.1:** In the first 12 months, weekly emails or phone calls were made by the Support Officer to in-School Champions to encourage implementation. In the second 12 months^#^, contact was made by the Support Officer to in-School Champions via email and phone according to the following high dose/low dose protocol matched to school practice uptake in the preceding term (e.g. Term 5 informs Term 6). As outlined in sub-strategy 3.4:  a. Schools adopting four or less practices should receive six emails or phone calls in the term.  b. Schools adopting five or more practices should receive seven emails or phone calls in the term. | None |
| **6.2:** Automated messages were sent each term via the program website to in-School Champions to prompt completion of teacher professional learning and online termly performance monitoring and feedback surveys. | The website automatically generated up to three emails per term to remind in-School Champions and PE teachers to complete professional learning modules. |
| **7. Implementation performance monitoring and feedback** |  |
| **7.1**: In-School Champion completes all termly surveys via the program website (PA4E1 Online). | Termly surveys were completed by in-School Champions via the Surveys and Progress Reports tab. |
| **7.2**: A feedback report is automatically generated and sent to in-School Champions via email | The website automatically generated feedback reports and sent these to registered in-School Champion users of the website. The website also housed copies of the surveys and progress reports. |
| **7.3**: A feedback report is automatically generated and sent to school Principals via email. | The website automatically generated feedback reports and sent these to registered Principal users of the website. |
|  |  |
|  |  |
| **Footnotes:**  * Only offered in the first 12 months of implementation support, not offered in the second 12 months^#^.  ^ Following team discussion, the team agreed that one or more logins per week would represent very high usage. At least one login per fortnight would represent a utility-style usage (i.e. used purposefully but not beyond its core function).  ^#^ 24 month program later extended to 26 months (modification made during program implementation, from 26-months, October 9th 2017 to December 20th 2019). The initial term (Term 4, 2017) was dedicated to supporting school planning, with implementation of the physical activity practices to commence in 2018. The implementation support intervention continued until the end of Term 3, October 2019. Over the support period there were incremental milestones for schools to embed physical activity practices into their usual business in 2018 and 2019. An additional 9th term of support beyond that described in the study protocol focused on sustainability of the practices into the next school year (2020), and included face-to-face professional development and development of a sustainability plan with the assistance of the Support Officer (school Term 4 end, Oct -December 20th 2019). The total length of the program was therefore 26 months (October 9th 2017 to December 20th 2019). | |

| **Supplementary File 1, Table 2:** Overview of the evidence-based PA4E1 program (physical activity practices) including standards required of program schools (essential elements) and additional desirable elements (28). Reproduced from (32). |
| --- |
| **Physical activity practices by Health Promoting Schools domain** |
| ***Curriculum, teaching and learning*** |
| 1. **Quality Physical Education (PE) lessons:**  - PE department used documented principles or guidelines for teachers to maximize PE quality, active learning time and student engagement in PE lessons (Program schools used the SAAFE principles- Supportive, Autonomous, Active, Fair, Enjoyable) (60). - Each PE teacher participated in peer observation of a practical PE lesson, at least once a year*. - *Desirable – peer observation feedback is against the department’s quality PE principles.* |
| 1. **Student physical activity (PA) plans:**  - All Grade 7 students developed a personal PA plan which included  1. personal goals to improve or maintain activity or fitness 2. actions and timelines to achieve goals and 3. progress monitoring  - Goals reviewed once within year - *Desirable – students in Grades 7-10 develop a personal PA plan*** |
| 1. **Enhanced school sport program:**  - The school delivered a short (10-12 weeks) structured PA Program designed to improve adolescents’ fitness and provide them with knowledge, motivation and skills to engage in a range of lifelong physical activities. - The program should be delivered to all students in at least one Grade between 7 and 10 (Program schools delivered the Resistance Training for Teens program to all of Grade 7 (61). |
| ***Ethos and environment*** |
| 1. **Recess/ lunchtime physical activity:**  - Supervised recess and/or lunchtime PA sessions offered to all students in Grades 7-10 at least three days per week. - PA equipment freely available to students at least three days per week at recess and/or lunch. - *Desirable - at least one organized recess or lunch activity per week targeting girls. Sessions promoted to students at least once per term.*      1. **School PA Policy or Procedure:**  - School developed a policy which included:  1. Provision of at least 150 minutes/week of MVPA during school time for all students in Grade 7-10; 2. Supportive practices to enhance all students’ PA (at least 3 of practices 1-4, 6-7 in this table)*** |
| ***Partnerships and services*** |
| 1. **Links with community physical activity providers:**  - School has at least three links that went beyond promotion of the provider (e.g. in newsletters) to involve an agreement, connection, partnership (e.g. out of hours sessions on school facilities, presentation by providers at school). - Links were designed to support ‘outside of school time’ activity. - Links were communicated to students and families at least once per term****. - *Desirable - at least one of the community links made were to promote free or low cost options in the community.*  1. **Communicating physical activity messages to all parents**  - All parents of students in Grades 7-10 were sent PA messages that were designed to increase parent knowledge, attitudes and support towards PA, at least once per term. (excludes messages about school events e.g. carnivals, or school sports timetables or results, or advertisements for community PA providers).**** |
