# Supplementary material for: Evaluating Digital Program Support for the Physical Activity 4 Everyone (PA4E1) School Program: Mixed Methods Study
Source: JMIR Pediatr Parent. 2021 Jul 26;4(3):e26690. doi: 10.2196/26690 (PMC8367175; doi:10.2196/26690)
Supplement: Multimedia Appendix 2 [file pediatrics_v4i3e26690_app2.docx]

| **Supplementary File 2** |
| --- |

| **Stage 1 checklist for the AMUsED Framework (1)** | | | | | | | | | | |  |
| --- | --- | --- | --- | --- | --- | --- | --- | --- | --- | --- | --- |
| **Familiarisation with the data – identifying variables** | | | | | | | | | | |  |
| Generic questions by data type | | | Intervention: Physical Activity 4 Everyone (PA4E1) | | | | Intervention: *Internet Dr* | | | |  |
| **1. Intervention characteristics. Data for intervention architecture and content.** | | | | | | | | | | |  |
| **1.1 Workflow. Intervention structure and expected participant interaction and navigation through the intervention.** | | | | | | | | | | |  |
| How many logins/sessions are available? | | | *Not structured as sessions, access intended for three reasons: 1. Ad hoc (All users, e.g. access resources); 2. To complete training modules (PE Teachers and School Champions only, released sequentially); 3. To complete termly surveys (School Champions only)*  *The following sections use the format 1,2,3 referring to these three components.* | | | | *Not structured as sessions, access intended during an RTI.* | | | |  |
| When are they available? | | | *1. Ad hoc material (e.g. resources, school case studies ‘school in focus’) available at every access, with some additional material uploaded over time.*  *2. All training modules available from the beginning (except 1 which was released later and was a summary video of the other 7)*  *3. Termly surveys made available each school term for 8 school terms.* | | | | *All content available at every access. Prompted logins at: baseline, 5 x 4-weekly interim questionnaires, & follow-up questionnaires. Then during illness and 48 hour follow-up.* | | | |  |
| Are new sessions released depending on time elapsed or task-completion? | | | *1. No*  *2. Professional learning videos released sequentially each term for first four terms on week 2 of each term.*  *3. Termly surveys are released during week 8 of 10 of each term, and are open for two weeks prior to feedback being given.* | | | | *No.* | | | |  |
| Are there limitations on the availability of the intervention? | | | No. | | | | *No.* | | | |  |
| Is the purpose of a session to collect self-report measures and/or use the intervention? | | | 1. No  2. No  3. Termly surveys are not an outcome measure, they are part of the implementation strategy “Performance Monitoring and Feedback”. An automated feedback report is sent to registered School Champions and Principals of that school after School Champion completes a survey. There are eight surveys running from Term 4 2017 to Term 4 2019. | | | | *Questions are asked after login to ascertain user’s purpose – either to use intervention for illness or to complete interim questionnaires.* | | | |  |
| When is the intervention considered to be finished? | | | The study finishes at the end of term 4 2019 (December 20^th^). | | | | *The study finishes after the follow-up questionnaire at 24 weeks. The intervention was finished when users no longer wished to access it.* | | | |  |
| What prompts are used to encourage usage (e.g. emails, texts, notifications) and when are they sent? | | | 1. No prompts  2. Prompts are sent to School Champions and PE teacher user accounts to ask them to complete training on Monday week 2,5 and 7 of each term.  3. Reminders to complete termly surveys are sent on Monday week 8 of each term via email, then Monday week 9 and then week 9 on Thursday for final prompt. Prompts contingent on non-completion. | | | | *Email prompts for 4 weekly data collection.*  *Additional emails are sent after logging in during illness to prompt repeat login after 48 hours.* | | | |  |
| Does the intervention contain ‘tunneled’ (compulsory) sequences of pages which users have to view to move forward? | | | 1. No  2. No  3. No | | | | *‘Doctor’s Questions’ component is tunneled as symptoms are assessed before providing illness management advice.* | | | |  |
| Are users able to select linked components they wish to view, and avoid others? | | | No. | | | | *There are 3 linked menu components available from the home page.* | | | |  |
| **1.2. Content. Content available within the pages of the intervention.** | | | | | | | | | | |  |
| What are the components available? | | | | As per the logic model, there are 6 of the 7 support strategies available on the website. These are:  1. Executive support – principals registered for PA4E1 Online website receive feedback reports on school progress.  2. In-School Champion – can access the website.  3. Professional learning – videos available with short quiz after each video for School Champions and PE teachers.  4. Resources – available via resources tab  5. Prompts – automatically generated based on user actions in the website e.g. not completing training modules will trigger a reminder.  6. Performance monitoring and feedback – surveys are available to complete on the website each term which generate automated feedback to the School Champions and Prinicpals (only registered users). | | | | *‘Doctor’s Questions’. Asks about symptoms & provides advice recommending either: self-management, phoning NHS Direct, or seeking immediate medical attention. ‘Treatment Options’. Advice on coping with symptoms: without medication, with medication, and boosting immune system. ‘Common Questions’. 2 sets of FAQs: ‘Ask the Internet Dr’ - medical questions about illness and treatment, ‘Common myths about Colds and Flu’ - general questions about illness beliefs.* | | | |
| What is the aim of each component and are they based on underlying theoretical constructs? | | | | The Behaviour Change Wheel and the Theoretical Domains Framework were used to develop the multi-component implementation support strategies. | | | | *Doctor’s Questions and Common Questions support users who are unsure if their symptoms are serious and whether they need medical treatment. Based on Leventhal’s Common Sense Model of Self-regulation of health and illness* [3] *to increase understanding of illness. Treatment Options supports management of distressing symptoms. Based on Bandura’s Social Cognitive Theory* [4] *to increase self-efficacy.* | | | |
| In what order is it anticipated the components will be used? | | | | No specific order. | | | | *Doctor’s Questions first so as to check symptoms.* | | | |
| What interactive features are available (e.g. forums, videos, printable information)? How long should they take to complete? | | | | Training videos  Discussion Forum  Printable Resources  Surveys with subsequent feedback reports | | | | *Videos available: Welcome video on home page: 1 minute, 23 seconds, glands: 48 seconds, meningitis/septicaemia: 1 minute, 1 second, video sinusitis: 30 seconds. Printable material available but log-data doesn’t record whether it was used.* | | | |
| Are all components/features available to all users throughout the intervention or are some tailored for specific times or users? | | | | School Champions receive everything.  PE Teachers receive everything except surveys and feedback reports.  Principals only receive basic information and feedback reports.  Admin staff only receive basic information. | | | | *Doctor’s Questions and Treatment Options are split into cough, sore throat, runny nose & fever.* | | | |
| Which pages are for collecting self-report measures or for administrative purposes | | | | None | | | | *Separate list available for content by individual page.* | | | |
| Are there specific pages to mark the start and end of sessions? | | | | *Home page is the first page visited.* | | | | *Home page is the first page visited.* | | | |
| Which pages contain BCTs (e.g. information, planning, feedback) and what are they? | | | | *See components listed previously* | | | | *See components listed previously* | | | |
| In which sessions are they available? | | | | *1. Ad hoc (All users, e.g. access resources) – Resources available*  *2. To complete training modules (PE Teachers and School Champions only, released sequentially); - Training available*  *3. To complete termly surveys (School Champions only) – Termly surveys available* | | | | *N/a* | | | |
| Can specific BCTs be identified on particular pages or groups of pages? How many groups are there? | | | | *See components listed previously.* | | | | *See components listed previously* | | | |
| Do any of the pages have response options to collect information in addition to baseline/follow-up measures? What data is collected? | | | | *Yes, termly surveys are an alternate measure of schools implementation of seven physical activity practices (primary outcome of trial).* | | | | *Information on symptoms is collected in Doctors Questions.* | | | |
| **2. Accrued data. Data collected during an intervention.** | | | | | | | | |  |  |  |
| **2.1. Self-report. Users’ self-reported responses collected across various stages of the trial.** | | | | | | | | |  |  |  |
| When are self-report questionnaires collected? | Termly Surveys each term and Talk-aloud usability study at 12 months. | | | | *Baseline, every 4 weeks up to 24 weeks, during illness and 48 hour follow up.* | | | |  |  |  |
| What demographic information is available (e.g. age, gender, education)? | School Champion: years teaching, number of school champions (turnover) | | | | *Baseline: Age, gender, qualification, household, smoker, alcohol, ethnicity.* | | | |  |  |  |
| Which measures are specifically related to the target behaviour and how often are they collected? | Termly – measuring schools implementation of the seven physical activity practices (primary outcome) | | | | *During illness, every 4 weeks & follow-up (see also external data): occurrence of illness and contact with all NHS.* | | | |  |  |  |
|  | | | | | | | | | | |  |
| Which measures of target behavioral determinants are collected and when? | | | None | | | | *Baseline & follow-up: health locus of control* [5]*, Krantz health opinion survey* [6]*, TPB* [1]*. When ill & 48hr: IPQ-R* [7]*, TPB* [1]*.* | | | |  |
| Are measures of health collected (e.g. conditions which may impact on target behaviour or are co-morbid) and psychosocial factors (e.g. anxiety, illness perception, motivation)? | | | None. | | | | *Baseline, when ill & 48 hour, & follow-up: (Physical) Mobility, self-care, usual activities, pain, anxiety/depression. Baseline (see also external data): health anxiety inventory* [8]*.* | | | |  |
| Are additional measures collected at follow-up (e.g. satisfaction, adherence)? | | | At 12 months – usability via Systems Usability Scale (2), Subjective Experience Engagement via User Engagement Scale (3) and Task completion via a set of task using Loop 11 tool (4) | | | | *Follow-up: patient enablement* [10]*, website satisfaction, problematic experiences of therapy scale* [11]*.* | | | |  |
| **2.2. Log-data. Information automatically collected through engagement with an intervention.** | | | | | | | | | | |  |
| What data is the software platform able to record? | | | Number of sessions (n), training modules completed (n/8), initiation of termly online surveys (binary), completion of online surveys (binary), discussion forum posts and replies, pages viewed (n), resource downloads, time per session, | | | | *Time and date, pages viewed & order, time spent on pages, self-report measures.* | | | |  |
| Are number, date and time of logins available by individual user? | | | Available via user type. | | | | *Yes.* | | | |  |
| Are individuals’ total durations of usage accessible? | | | Yes | | | | *Needs to be extracted.* | | | |  |
| Are the number and time of usage prompts recorded? | | | Yes. Number of session and average length of sessions. | | | | *Overall scheduled timings for emails are available, but not sent times by individual.* | | | |  |
| Are there details for which pages were viewed, the sequential order and time spent viewing? | | | *Pages viewed yes, but not order.* | | | | *Yes.* | | | |  |
| **2.3. External data. Data collected independently but alongside intervention usage.** | | | | | | | | | | |  |
| How and where is the data collected? | | | Talk-aloud survey and usability test.  Focus Groups with School Champions  Interviews with PE Teachers and Principals  Surveys with Head PE teacher (collecting Primary trial outcome), students, and School Champion  Student accelerometry (secondary trial outcome) | | | | *Hand collected by the research team from users’ GP notes.* | | | |  |
| What data is collected? | | | See intervention protocol (5) and process evaluation protocol (6). | | | | *Number of GP visits for RTI during trial, for year prior to trial, and co-morbid illnesses. Antibiotic prescriptions for RTI.* | | | |  |
| Which of these measures relate to or may impact on the target behaviour? | | | All of them may relate to the target behaviour. | | | | *Number of GP visits for RTI during trial, for year prior to trial, and co-morbid illnesses.* | | | |  |
| **3. Contextual data. Data indirectly related to the running of the intervention which may be influential over usage and analysis.** | | | | | | | | | | |  |
| **3.1. External factors. Structures and events which may influence participation in the intervention.** | | | | | | | | | | |  |
| How are users recruited to the intervention? | | | Schools recruited via Principal email. | | | | *Recruited via GP.* | | | |  |
| Did any specific large-scale events, with the potential to impact on the intervention, occur during the period of the intervention? | | | No, though we do have a context monitoring log that lists some smaller events. | | | | *N/a* | | | |  |
|  | | |  | | | |  | | | |  |
|  | | | | | | | | | |  |  |
| **3.2. Previous theory and findings. Results of behavioral analyses carried out during intervention development (e.g. logic models), and analyses of clinical outcomes if available.** | | | | | | | | | |  |  |
| What are the hypothesized mechanisms of the intervention (e.g. as specified in the intervention’s logic model)? | | The implementation support strategies are delivered to schools and this results in schools implementing the seven physical activity practices, which results in the schools being more physically active. | | | | *Users with low understanding of their symptoms will be less likely to consult their GP having viewed Doctors Questions and Common Questions. Users with low self-efficacy in their ability to self-manage their illness will be less likely to consult their GP having viewed Treatment Options.*  *Level of anxiety may be associated with usage.* | | | |  |  |
| Which factors are identified as important in qualitative research, and can they be related to the variables collected in the trial? | | N/A | | | | *N/a* | | | |  |  |
| Which variables are identified as relating to outcomes (e.g. behavioral determinants, theoretical constructs, health factors)? | | N/A | | | | *Analysis of the data from the RCT focused on the outcomes of GP contacts and antibiotic use* [13]*. No analyses were carried out for usage, behavioral determinants or personal characteristics.* | | | |  |  |
|  | |  | | | |  | | | |  |  |

**Stage 2 checklist for the AMUsED Framework**

| **Selecting usage variables and generating research questions** | | | | |
| --- | --- | --- | --- | --- |
| Generic questions | Intervention: *Physical Activity 4 Everyone (PA4E1)* | | Intervention: *Internet Dr* | |
| **1. Descriptions of usage variables. Which usage variables are relevant to the intervention and in which format (e.g. number of users/sessions, duration, percentage of total, dichotomous)?** | | | | |
| Completing intervention/trial period (stage 1; 1.1 & 2.2). | Number of users (School Champions, PE teachers, Principals and School admin staff) registering for PA4E1 Online (website).  Number of users downloading resources.  Number of users accessing a particular page.  Time spent on pages.  Number of School Champions and PE Teachers completing training modules.  Number of School Champions completing termly surveys. | | *Number of users completing follow-up measures at 24 weeks. Number of users ill during trial period (logged in during illness, self-report at 4 week measures, from GP notes). Total number of pages viewed across the trial and pages with the greatest number of views.*  *Total time spent on pages.* | |
| Logins or sessions where the intervention was accessed (stage 1; 1.1 & 2.2). | N/A | | *Purpose of login: to complete baseline, 4 weekly measures or manage illness. Content viewed, number of pages and time spent when using intervention during illness.* | |
| Date of login and usage. | Session dates for each user groups. | | *n/a* | |
| Time of day of login and usage. | N/A | | *Proportion of usage outside vs during GP hours.* | |
| Days/weeks of usage (stage 1; 1.1 & 2.2). | Number of sessions website was accessed. | | *Number of times intervention content was accessed.* | |
| Response to prompts/notifications (stage 1; 1.1 & 2.2). | Number of sessions by School Champions at the time of termly survey prompt. | | *Number of users who logged in to illness follow-up at 48 hour.* | |
| Features/menu components used (stage 1; 1.1, 1.2 & 2.2). | Number of users viewing each page  Number of downloads of resources  Most viewed pages.  Most downloaded resources.  Number of training modules completed. | | *Number of users who viewed each of the linked menu components. Component most frequently viewed first. Doctors Questions: Dropout by page. Number of users moving back and forwards through pages. Split of users by symptom. Number of users who saw the advice pages, and type of advice given. Treatment Options & Common Questions: Number of pages viewed. Time spent in this component. Number of users who viewed each of the treatment options. Split of users by symptom.*  *Number of users viewing each video. Time spend on the video.* | |
| Revisiting components/features (stage 1; 1.1, 1.2 & 2.2). | *N/A* | | *Number of users re-visiting each component and frequency of revisits. Number of re-visits during illness. Number of users revisiting components with the same/different symptoms. Number of users who login when ill more than once.* | |
| Type of content/BCTs used (excluding administration pages) (stage 1; 1.2 & 2.2). | *See features/menu components* | | *See features/menu components* | |
| Completing ongoing measures (stage 1; 2.1 & 2.2). | *Number of school champions completing termly surveys.* | | *Number of users completing monthly measures.* | |
| External device usage (stage 1; 2.3). | *Access via mobile vs laptop* | | *N/a* | |
| **2. Relationships between usage and participant characteristics. Are user’s demographic, physical or psychosocial characteristics at baseline related to intervention usage?** | | | | |
| Are any characteristics at baseline related to usage? | N/A low sample sizes | | *Is having a co-morbid illness related to usage? Is age associated with different patterns of usage? Is level of anxiety at baseline associated with different patterns of usage?* | |
| Are any contextual factors related to usage (stage 1; 3)? | N/A | | N/a | |
| Do high/low users differ by other usage factors? | Do type of users (i.e. School champion have most access, then Pe Teachers, then Principals and finally school Admin staff) use the intervention differently? I.e. different pages, number of sessions, number of downloads | | *Is logging in when ill related to content previously viewed? Do users who self-report GP visits use the intervention differently to users who visited their GP but didn’t report it?* | |
| **3. Relationships between usage, behavioral determinants, and target behaviors. Which usage variables are associated with follow-up measures for target behaviour and behavioral determinants? Which usage variables help explain changes in behaviour across the intervention?** | | | | |
| Are baseline measures for behavioral determinants/target behavior related to usage? E.g. Is the number of days the intervention is used for related to a behavioral determinant? Do users with low target behavior spend less time on the intervention? | N/A | | *Is the number of GP visits during year prior to trial related to usage? Are measures for health locus of control [*2]*, Krantz health opinion survey* [3]*, or TPB* [4] *attitudes/norms/intentions at baseline related to usage? Are measures for the IPQ-R* [5]*, TPB* [4] *beliefs/intentions at first login during illness related to usage during the period of illness (e.g. viewing content, completing 48hr follow-up).* | |
| Which usage variables are related to behavioral determinants/target behaviors and at follow-up? E.g. Do users who view a group of pages | Did 24 month CATI practice uptake score, and termly surveys from term 1-8, relate to usage/sessions? | | *Is usage related to GP appointments? Is logging in when ill related to GP appointments? Is usage associated with the amount of times a user is ill* | |
| containing a specific BCT score higher/lower for the associated behavioral determinant? Is completing/not completing a particular component associated with target behavior at follow-up? Is the time spent on a session related to target behavior? | |  | | *during the trial? Are measures for health locus of control* [2]*, Krantz health opinion survey* [3]*, or TPB* [4] *attitudes/norms/intentions at follow-up related to usage? Are measures for the IPQ-R* [5]*, TPB* [4] *beliefs/intentions at 48hr related to viewing content during illness?* |
| Is usage associated with measures for acceptability/satisfaction at follow-up? E.g. Are high levels of satisfaction associated with accessing more pages? Do users with low satisfaction spend less time using external devices? | Do usability and engagement scores (2, 3) from 12 month talk-aloud usability and engagement survey relate to number of session in the website? | | *Are scores for patient enablement* [7]*, website satisfaction, or problematic experiences of therapy scale* [8] *related to usage?* | |
| Do users who report positive changes in behavioral determinants/target behavior from baseline to follow-up use the intervention differently to those who do not? | N/A | | *Did users with positive changes for measures for health locus of control* [2]*, Krantz health opinion survey* [3]*, or TPB* [4] *attitudes/norms/intentions use the intervention differently? Did users who reported increased positive beliefs at 48hr illness follow-up view different content during illness?* | |
| Are relationships between usage and outcome measures moderated by demographic, psychosocial or health factors? | N/A | | *Does anxiety moderate relationships between usage and GP visits? Does age moderate the relationship between usage and GP visits?* | |
| What level of usage is necessary for ‘effective engagement’? | Did users need to access the videos in order to change their behaviour? | | *Did users need to see a specific amount or component of content, at a certain time in order to change their behaviour?* | |

**Stage 3 checklist for the AMUsED Framework**

| **Preparation for analysis** | | | | |  |
| --- | --- | --- | --- | --- | --- |
| Generic questions | Intervention: *Physical Activity 4 Everyone (PA4E1)* | | Intervention: *Internet Dr* | |  |
| **1. Resources** | | | | |  |
| What is the timeframe for completing the analyses? | July 2020 | | *Oct 2018.* | |  |
| What resources are needed? E.g. additional research time, expertise | Existing research team is diverse enough and has statistician support. | | *Support with analyses, confounding variables (e.g. co morbid illnesses), and moderators. Support with visually exploring and extracting data.* | |  |
| Is a plan of analysis already available? How does the analysis plan developed using the framework compare to that plan? Are changes or updates needed? | No. | | *Primary analyses have already been carried out (see stage 1, 3.2), no further plan is available.* | |  |
| Is ethical clearance in place to carry out usage analyses? | Yes. | | *Yes.* | |  |
| **2. Selecting types of analysis and analytical software** | | | | |  |
| Will the usage data be triangulated with qualitative data? | Yes. | | *No.* | |  |
| What analytical tools are available? |  | | *SPSS and LifeGuide Visualisation Tool (LVT).* | |  |
| Is there sufficient statistical power to answer the planned research questions? | *None of the analyses using schools as the unit (n=24), or school champions or school principals will be. However, analyses with PE teachers may be?* | | *Analyses using whole intervention group are sufficiently powered, sub-group analyses will not be.* | |  |
| Can the selected measures of usage be analyzed using the available tools? Is bespoke software necessary (e.g. visualisation techniques)? |  | | *Sequence of use of components and movement through Doctors Questions will need to be analyzed using LVT.* | |  |
| **3. Data preparation** | | | | |  |
| When is the data available? | Now. | | *Now.* | |  |
| Is the data raw or has it been used/cleaned previously? | Raw log-data. | | *Raw log-data.* | |  |
| How many datasheets are there? Will these need to be amalgamated? | All available from Google Analytics, just need to download what’s relevant for the research questions. Codes from Google Analytics can be cross-referenced to find the individual users demographic information. | | *5 datasheets: 4 log-data sheets, GP notes. LVT: Session details must contain important self-report measures from user data. Copy primary outcome measures, previous behaviour and co morbid* | |  |
|  |  | | *illnesses from GP notes. SPSS: all data for analysis must be available on one data sheet. Relevant measures from GP notes will be copied to session details, along with usage variables from user data and page durations.* | | |
| Is the data structured to work with the tools available? What formats are the datasheets in (e.g. excel, .csv) and will they need converting for analysis? | | Yes, data can be made available as descriptive statistics. | | *Datasheets are compatible with SPSS and LVT. Log data (session details, page flow, page durations, user data) is excel worksheet. These need to be converting to .csv for LVT. GP notes are SPSS.* | |
| What preparation does the data need (e.g. cleaning, anonymizing)? | | The descriptive statistics need running. | | *Data needs cleaning.* | |
| Are all variables readily available or will they need extracting/transforming/recoding? | | Some tidying and cleaning will need to occur, but most are ready. | | *User data datasheet has a row of data per login so that a user who has logged in 7 times will have 7 rows of data. However, session details datasheet has only 1 row per user. Data must be transformed into 1 row in order to copy across. Totals for time spent and pages viewed will need extracting.* | |
| Is the data in the right format to answer the research questions? Will it need adapting (e.g. continuous variables changed to categorical)? | | For the triangulation between qualitative and quantitative, the data will be in the right format. | | *Measures of behavioral determinants are measures as continuous variables. For comparisons of usage by high/low groups these* | |

1. Miller S, Ainsworth B, Yardley L, Milton A, Weal M, Smith P, et al. A Framework for Analyzing and Measuring Usage and Engagement Data (AMUsED) in Digital Interventions: Viewpoint. J Med Internet Res. 2019;21(2):e10966.

2. J B. SUS: A quick and dirty usability scale. Redhatch Consulting, Reading.; 1996.

3. O’Brien HL, Cairns P, Hall M. A practical approach to measuring user engagement with the refined user engagement scale (UES) and new UES short form. International Journal of Human-Computer Studies. 2018;112:28-39.

4. The remote usability testing tool Loop11.

5. Sutherland R, Campbell E, Nathan N, Wolfenden L, Lubans DR, Morgan PJ, et al. A cluster randomised trial of an intervention to increase the implementation of physical activity practices in secondary schools: study protocol for scaling up the Physical Activity 4 Everyone (PA4E1) program. BMC Public Health. 2019;19(1):883.

6. McLaughlin M, Duff J, Sutherland R, Campbell E, Wolfenden L, Wiggers J. Protocol for a mixed methods process evaluation of a hybrid implementation-effectiveness trial of a scaled-up whole-school physical activity program for adolescents: Physical Activity 4 Everyone (PA4E1). Trials. 2020;21(1):268.
