# Supplementary material for: Evaluating Digital Program Support for the Physical Activity 4 Everyone (PA4E1) School Program: Mixed Methods Study
Source: JMIR Pediatr Parent. 2021 Jul 26;4(3):e26690. doi: 10.2196/26690 (PMC8367175; doi:10.2196/26690)
Supplement: Multimedia Appendix 3 [file pediatrics_v4i3e26690_app3.docx]

| **Supplementary File 3** |
| --- |

# In-School Champion Think-aloud survey and usability test

# Questions

## 1. Practice question: I like driving red cars. (Please elaborate by talking aloud).

An example of a verbal response could be:

"I don't drive red cars because they are not cool."

Or conversely,

"I love red cars because they are cheap, reliable and easy to drive"

*Response Options*

*Strongly Disagree | Disagree | Neutral | Agree | Strongly Agree*

2. Navigate to where you would post a discussion comment. You do not need to post anything. Also, don't forget you can elaborate on how easy or hard this was, as well as more general comments at any time, by talking into the microphone. Once you've completed the task, click 'Task Completed' (or 'Abandon Task' if you can't complete the task).

*Response Options*

*Task Completed | Abandon Task*

3. From the previous task: The discussion forum was easy to find (Please elaborate by talking aloud before clicking next).

*Response Options*

*Strongly Disagree | Disagree | Neutral | Agree | Strongly Agree*

## 4. In a hypothetical scenario, you are unsure how to form community links. Using PA4E1 Online, please navigate to find this information. Also, don't forget you can elaborate by talking into the microphone.

### *Response Options*

*Task Completed | Abandon Task*

## 5. From the previous task: the information was easy to find. (Please elaborate by talking aloud)

*Response Options*

*Strongly Disagree | Disagree | Neutral | Agree | Strongly Agree*

## 6. In a hypothetical scenario, your Support Officer has prompted you to encourage staff to conduct SAAFE observations. Please navigate to where you would log this information. Also, don't forget you can elaborate by talking into the microphone.

### *Response Options*

*Task Completed | Abandon Task*

## 7. From the previous task: the information was easy to find. (Please elaborate by talking aloud)

*Response Options*

*Strongly Disagree | Disagree | Neutral | Agree | Strongly Agree*

## 8. I am aware of all the components of PA4E1 Online and how these support the program. (Please elaborate by talking aloud).

*Response Options*

*Strongly Disagree | Disagree | Neutral | Agree | Strongly Agree*

## 9. This is an open task for you to navigate around the site freely, to any page you wish, and make comment (talk-aloud into the microphone) about what you might change or add-in if you were to redesign the site.

*Response Options*

*Task Completed | Abandon Task*

## 10. I am aware of all the main resources on the website. (Please elaborate by talking aloud).

*Response Options*

*Strongly Disagree | Disagree | Neutral | Agree | Strongly Agree*

## 11. The discussion forum is a good mode of communication for the PA4E1 program. Its content, complexity, delivery and credibility make it acceptable for me to use. (Please elaborate by talking-aloud).

*Response Options*

*Strongly Disagree | Disagree | Neutral | Agree | Strongly Agree*

## 12. With regards to PA4E1 Online only, indicate how strongly you agree or disagree.

(Please elaborate by talking-aloud).

1. I think that I would like to use this frequently
2. I found this unnecessarily complex
3. I thought this was easy to use
4. I think that I would need the support of a technical person to be able to use this
5. I found the various functions were well integrated
6. I thought there was too much inconsistency
7. I would imagine that most people would learn to use this very quickly
8. I found this very cumbersome to use
9. I felt very confident using this
10. I needed to learn a lot of things before I could get going with this

*Response Options*

*Strongly Disagree | Disagree | Neutral | Agree | Strongly Agree*

## 13. In a hypothetical scenario, the PA4E1 program is to be rolled out to all schools state-wide (NSW). Is PA4E1 Online, in its current form, a suitable delivery mode? (Please elaborate by talking out loud).

## *Response Options*

## *Yes, because.... (please elaborate by talking-aloud) | No, because... (please elaborate by talking-aloud)*

## 14. Please indicate how strongly you agree or disagree with the following statements

1. PA4E1 Online meets my approval.
2. PA4E1 Online is appealing to me.
3. I like PA4E1 Online.
4. I welcome PA4E1 Online.
5. PA4E1 Online seems fitting.
6. PA4E1 Online seems suitable.
7. PA4E1 Online seems applicable.
8. PA4E1 Online seems like a good match.

*Response Options*

*Strongly Disagree | Disagree | Neutral | Agree | Strongly Agree*

## 15. In a hypothetical scenario, you are a decision maker within NSW Ministry of Health, NSW Department of Education or your Catholic Schools Office, and you are rolling out PA4E1 across the state. Should online delivery remain a part of PA4E1?

*Response Options*

*Yes, because....* *(please elaborate by talking-aloud) | No, because...* *(please elaborate by talking-aloud)*

16. In a hypothetical scenario, PA4E1 Online is being upgraded for delivery across NSW. As part of this, Support Officers will now be on hand during working hours (9am-5pm) via the website. The website will have a video, audio and text chat features that allow video-to-video calling. This means you can get support quickly with the project. What are your thoughts?

*Response Options*

*I LIKE the idea because....* *(please elaborate by talking-aloud) | I DISLIKE the idea because...* *(please elaborate by talking-aloud)*

## 17. Please indicate how strongly you agree or disagree with the following statements

1. PA4E1 Online meets my approval.
2. I lost myself in this experience.
3. I was so involved in this experience that I lost track of time.
4. I blocked out things around me when I was using PA4E1 Online.
5. When I was using PA4E1 Online, I lost track of the world around me.
6. The time I spent using PA4E1 Online just slipped away.
7. I was absorbed in this experience.
8. During this experience I let myself go.
9. I felt frustrated while using this PA4E1 Online.
10. I found this PA4E1 Online confusing to use.
11. I felt annoyed while using PA4E1 Online.
12. I felt discouraged while using this PA4E1 Online.
13. Using this PA4E1 Online was taxing.
14. This experience was demanding.
15. I felt in control while using this PA4E1 Online.
16. I could not do some of the things I needed to do while using PA4E1 Online.
17. This PA4E1 Online was attractive.
18. This PA4E1 Online was aesthetically appealing.
19. I liked the graphics and images of PA4E1 Online.
20. PA4E1 Online appealed to be visual senses.
21. The screen layout of PA4E1 Online was visually pleasing.
22. Using PA4E1 Online was worthwhile.
23. I consider my experience a success.
24. This experience did not work out the way I had planned.
25. My experience was rewarding.
26. I would recommend PA4E1 Online to my family and friends.
27. I continued to use PA4E1 Online out of curiosity.
28. The content of PA4E1 Online incited my curiosity.
29. I was really drawn into this experience.
30. I felt involved in this experience.
31. This experience was fun.

*Response Options*

*Strongly Disagree | Disagree | Neutral | Agree | Strongly Agree*

## 18. In a hypothetical scenario, PA4E1 Online is being upgraded. It's both enhancing existing features and getting new features. What would you recommend as changes?

### *Response Options*

*I would change something…(please elaborate by talking-aloud) | I wouldn't change anything…(please elaborate by talking-aloud)*

## 19. If you have any additional comments, please say now by talking-aloud before you close the survey.

### *Response Options*

### *Finish survey.*
