# Supplementary material for: Evaluating Digital Program Support for the Physical Activity 4 Everyone (PA4E1) School Program: Mixed Methods Study
Source: JMIR Pediatr Parent. 2021 Jul 26;4(3):e26690. doi: 10.2196/26690 (PMC8367175; doi:10.2196/26690)
Supplement: Multimedia Appendix 4 [file pediatrics_v4i3e26690_app4.docx]

| **Supplementary File 4**  **Triangulation Matrix** | | | |
| --- | --- | --- | --- |
| **The engagement framework has been utilized from Perski et al (1) focusing on the ‘Deliver’ and ‘Content’ components.** | | | |
| **Themes** | **Quantitative Data – Descriptive Statistics** | **Qualitative Data – Example Quotes** | **Convergence label (agree, slightly agree, silence, dissonance)** |
| **DELIVERY (Aesthetics/design, challenge, complexity, control features, credibility features, ease of use, familiarity, guidance, interactivity, message tone, mode of delivery, novelty, narrative, personalization, professional support features)** | | | |
| **META-THEME 1:**  **The website was an acceptable and appropriate delivery mode. Usability of the website was high.** | Most School Champions agreed or strongly agreed that the website was acceptable and appropriate. In-School Champions (n=13) Acceptability of Intervention measure was 4.52 (0.04) out of 5. In-School Champions (n=13) Intervention Appropriateness Measure was 4.46 (0.0) out of 5.  Additionally, in-School Champions (n=13) overall User Engagement Scale score (mean (SD)) was 3.4 (0.7) out of 5 (32). Individual dimension scores from the User Engagement Scale (mean(SD)) were 4.2 (0.1), 3.7 (0.5). 3.4 (0.3) and 2.4 (0.3) for Aesthetic Appeal, Reward Factor, Perceived Usability and Focused Attention, respectively.  **Usability**  The majority of In-School Champions (n=13) completed the three navigation tasks successfully (74% of tasks). The average time to task completion was 71 seconds. The discussion forum and community links tasks had higher success rates (85% and 92% respectively) than the SAAFE lesson observation task (46%). Time to complete each task were 91, 66 and 55 seconds for discussion forum, community links and SAAFE observation tasks, respectively. No tasks were abandoned.  Additionally, In-School Champions (n=13) overall Systems Usability Scale score was 77.7, which corresponds to a ‘good’ website within the ‘acceptable’ range of the Systems Usability Scale (31, 37). | “Yeah, I think the online portal in its current form is kind of suitable, I think a few minor changes and adjustments would make it better.”  “Um, it's set out very clearly, easy to use, easy to access, um, and is updated relatively frequently.”  “…the online portal is essential with implementation of the program. You couldn't do it without it. It's your go-to... go-to resource.”  “So, we all know that teachers are the time poor people of the world. Um, so definitely having an on-line, um, mode is very, very suitable.”  **Usability**  “…the portal is very easy to- to navigate.”  “My staff and I have found it really user-friendly”  “I think the site's very easy for me. Clearly set out, and most people that I know would easily be able to navigate and find answers to any questions they might have. And it's not a boring site with too many words on each page, it's well set out.”  “Um, as I said, I'm not great at technology, and I found it fairly easy to use.”  “It was easy to find [the information on community links].”  “Um, I think this section of the portal is very easy to- to navigate.” | Agree |
| **The website complimented other delivery modes, but in-School Champions preferred the other delivery modes (Support Officer and face to face).** | Schools were contacted (and made contact) through other modes of delivery. For example, contact logs indicate 18/24 schools received a face-to-face contact with their Support Officer at least once a term in the first 12 months. In the second 12 months, this dropped to 8/24 schools. However, weekly emails or phone calls between Support Officers and in-School Champions occurred in 16/24 schools during the first 12 months, but this increased to 22/24 schools in the second 12 months.  The two face-to-face workshops were well attended. 23/24 School Champions attended the first workshop at the start of the program and 22/24 attended the second workshop at mid-point. | “So, if I wasn't to have a support officer, if [Support Officer’s name] wasn't to, kind of, be easily contactable and not visiting the school regularly, then yeah, I probably would use the online portal more.”  “It's [the website], it is probably nearly everything there that you need. And, if there's not, then I can always ask [my Support Officer] [name].”  “You've obviously got your support officer as well [as the website], but it's good just to have this, um, this portal”  “Yes [I like the website], but it needs to be accompanied by other things like face-to-face as well.”  “Um, but yeah, I just think at the moment we're [School Champions] all relying on our support officers to answer our questions rather than posting them in the discussion forum or potentially looking for all the resources in the portal [website].”  “Yes, to some degree [the website is a suitable mode of delivery of PA4E1], but also I think the face-to-face workshops work well and allows you to... brainstorm some ideas outside of your school.”  “I think it [the website] is the most valid and useful way to get information out quickly um, to those people, um running PA4E1. Um it makes sense to have an online portal where people can go find um relevant and quality information examples, as discussed earlier. Um I definitely think it should remain as one of our main delivery um ah or sources of delivery for information for those running PA4E1.”  “…it is an invaluable experience to meet, um at a common place um, where you can actually roll out um, ah the initial delivery um, and training um, for the first time that people are running PA4E1. However, as said before, um online delivery should remain part of this program.”  “I do very much appreciate our support people. I do very much appreciate the meetings face to face. I don't think that the program would be as effective without these opportunities.”  “We also did our RT for Teens training together, and I found this also really, really beneficial. Obviously, you can't do this type of training on-line.”  “…probably the majority, maybe 70-75% should be on-line delivery. But there's still merit and a need for, um, hands-on, face to face delivery.”  “But I guess, he [Support Officer] kind of plays... He's I guess the easier version.”  “But if he [Support Officer] wasn't there I think all the answers you would need would be on the portal.”  “If you got rid of the support officer or maybe reduce the amount of times that you will see him or something like that. Then, I think, the online portal would kind of be even more necessary and useful.”  “I find, found my Support Officer, [Name], has, um, answered straight away anyway.”  “I like Physical Activity Online [the website]. Yes, I do 'cause it means you can access resources a- at any time.”  “teachers are often not being able to get this extra, sort of, um, work, or professional learning done during their actual hours of being at school. So I often access the website from home, after hours, on the weekend.” | Agree |
| **The website was used as a ‘utility’ delivery mode (i.e. used only when required, not as the first delivery mode of choice).** | The mean number of sessions per term for School Champions was 6.3 (SD: 2.0) and for PE Teachers is 0.8 (0.4). This means School Champions logged in on average every two weeks of the school term. | “To be honest I don't think I've used PA4E1 online, probably to its full potential.”  “I'm aware of most kind of, components of it, but there are things I'm not [aware of]...”  “I think I am aware of the main resources, I think there are resources on here but, there are probably I'm not aware of and that would be beneficial.”  “I suppose it just takes a little bit of kind of time to…have a look around because um, yeah it's a new thing.”  “Um, I didn't even realize this was all here [while navigating around the resources section of the website]. I hadn't seen that before, 'cause I haven't needed to look.”  “Um, yeah well obviously I haven't really used this [pointing at the resources section] as much as I should've. I have used some of these [resources] though, but I must have just kind of forgotten where I'd got them from um, in the past.”  “Face-to-face gives you more accountability.”  “[referring to professional learning]…I find that staff do just do the tic- tick through [not watching the video, but completing the quiz questions].”  “Yes [the website is appealing], 'cause you get to go back to it [the website], but you need some face-to-face stuff”  Yes [I like the website], but it needs to be accompanied by other things like face-to-face as well. | Agree |
| **If the program were delivered state-wide, the website would be useful, though embedding within other systems that teachers already used may be helpful.** | All in-School Champions (n=13/13) agreed that online delivery of the PA4E1 program should remain.  The majority of in-School Champions (n=12/13) suggested the PA4E1 website would be a suitable mode of delivery in its current form should the PA4E1 program be rolled out to schools state-wide. | “I do think it is quite suitable um, to roll out in its current form if there was to be no changes.”  “The more people that are on it [the website] as well, I think the more discussion that would kind of, be generated around there [the discussion forum].”  “…it is another platform that we need to access in order to implement our teaching.”  “It's a requirement that all department teachers complete their, uh, proficiency level and in order to do that, we need so many hours [and login to a different website to do this]. So, if there was a link to ... and I'm not sure if it does do this or not. But, if there was a link in the safe model modules, um, to the professional teaching standards, uh, or other links to curriculum specific learning, I think that it would be a resource that teachers would use more widely. So links to the syllabus, uh, and that of thing.” | Slightly Agree |
| **Mixed reactions towards the possible addition of a website chat feature with a Support Officer available to video call during business hours.** | The majority of in-School Champions (n=11/13) liked the idea that Support Officers would be available during working hours for support within the PA4E1 website via video, audio or text chat features to support School Champions. | **Supportive**  “t's great, because whenever you can squeeze it in in your free period there's someone there to talk to you about so you don't have to wait until the champion or someone in the know is available to help you, you've got someone at your core online, that's fantastic.”  “Um, yeah, I like this idea [having a chat feature embedded in the website], I guess we... We'll write an email at you or you have pretty good access to your support officer. Um, they're pretty reliable, they're very quick to reply. Having text chats and things like that on other websites, that could be very handy. Um, in fact you can get- you can have instant chat and things like that. And if you are having trouble navigating, um, online, that would be beneficial.”  **Neutral**  “I like the idea of the support offices being on hand. Uh, really like meeting face to face as well, but I know that um, that that's really time consuming for the Support Officers. Uh, they lose a lot of hours traveling, get to more of the remote parts of the state. I think it would be good if you could have something a little bit in between. Um, that, if there's no other option, then I think this definitely a good way to go.”  “I mean it might ease the load on the support officers having to kind of travel everywhere, 'cause then you have that option to sit and talk them like you would face-to-face. Um, but yeah I mean I like it but I- I- I don't dislike it but I don't know if it's hugely necessary.”  **Non-supportive**  “I dislike because I would prefer face-to-face. Email just works just as well, and a phone call if necessary.”  “Ah, I do think there's, there's still lots of value just in that simple text or a simple post to um, our Facebook site um, where other um, could answer a quick question, a common question or even just a email.”  “I'd just rather call the support officer via phone, or just email. I dunno if I'd go to the website and use that video, audio, text feature. You have to pay more for that on the website, I think I'd save my money and put it somewhere else.” | Slightly agree |
| **The discussion forum wasn’t used, but School Champions reported it could be potentially useful, perhaps if delivered differently.** | There were three discussion forum posts made by Support Officers to generate content. There were zero discussion forum posts by School Champions during the entire program.  **Facebook**  A Facebook group was established on 07/11/18. During the whole implementation of the program, there were 10 posts by the PA4E1 team (Support Officers and Staff) and 12 posts by School Champions in the Facebook Group that was established as a result of feedback from School Champions while at a face-to-face workshop. | “the discussion forum could be good and um, I just think the lack of discussion on there has made it, I guess, not beneficial for the school champions but that's as much for us to blame because we haven't been posting on there and we haven't been using on it.”  “Yep. It's…a little bit limited.”  “Discussion forums are always handy, but it appears that no one is actually using it. Is this something that could be eliminated or changed to some degree?”  “I haven't posted anything yet.”  “I've not really used it [the discussion forum] to be fair.”  “The more people that are on it as well, I think the more discussion that would kind of, be generated around there.”  **Should be delivered differently within the website**  “Um, I think this was mentioned at the training day, the suggestion that a discussion forum could pop up on the front page…so, it was the, kind of, first thing you saw, and the first thing that popped up, um, whenever you went there.”  “So I think the discussion forum on the home page would be beneficial, and you'd see it straight away and be able to click on anything that you find relevant to your school.”  “Ah, to be honest I have hardly used the um, discussion forum. I do believe they are um, great ways to communicate.”  **Should be delivered via Facebook**  “Um, I think the newly formed ah, PA4E1 group on Facebook might be a little bit more relevant um, and a little bit more widely used. Um, due to our, our um, use of Facebook and we tend to be on there a little bit more.”  “I don't think that the discussion forum is a good mode of communication. It appears that no one's really using it. Perhaps a different form of social media such as Facebook, which is happening, that more people use.”  “I think the discussion forum is actually great. Uh, I would use it except for the fact that almost nobody does, so then I would doubt that there would be, putting the question on there. People said this already, and we opened the Facebook group, and I-I don't think many people use that either, so, um, I'm not sure what's the best discussion forum place.”  “I like the idea of the Facebook program…because um, it's a little bit more kind of I suppose in most people's lives in some way just on a phone. Whereas coming into this [discussion] forum [pointing at discussion forum with cursor], you came in, you have to log into the site and then click through to the forum. It's not like a little notification or something.”  “the portal is not something I log onto everyday. As we all kind of said, like, everyone goes onto Facebook and things like that. So, that's where- and you get notifications that pops up on your phone, where often I don't know that things have been posted in the discussion forum, until I come back on, and often that's quite a while after they were post it.” | Agree |
| **CONTENT (Behaviour change techniques: feedback, goal setting, reminders, rewards, self-monitoring, social support features)** | | | |
| **META-THEME 2: The website content was generally acceptable and appropriate, with a few specific suggestions for improvement.** | Most in-School Champions agreed or strongly agreed that the website was acceptable and appropriate. In-School Champions (n=13) Acceptability of Intervention measure was 4.52 (0.04) out of 5. In-School Champions (n=13) Intervention Appropriateness Measure was 4.46 (0.0) out of 5. | “Yeah, I think the online portal in its current form is kind of suitable, I think a few minor changes and adjustments would make it better.”  “…the online portal is essential with implementation of the program. You couldn't do it without it. It's your go-to... go-to resource.” | Agree |
| **A lack of notifications (or prompts) to highlight new things in the website reduced return traffic to the website.** | None. | “…sometimes new things just pop up that I didn't really realize were there”  “…the portal [website] is not something I log onto everyday. As we all kind of said, like, everyone goes onto Facebook and things like that. So, that's where- and you get notifications that pops up on your phone, where often I don't know that things have been posted in the discussion forum, until I come back on, and often that's quite a while after they were posted.”  “Maybe also having some form of like, notification so in the top right hand corner…so that you know, like, you can see new…things that are kind of happening...”  “one thing I might change is that, notifying you when new modules come out because I know, as the staff, we've completed most of the modules to start with, and then a few more, I think it was one more the Summary of Safe got added, right at the end, so you can see the others were completed on the 14th or around those dates. Um, and when the last one got added it- We weren't aware of that. Wasn't until [Support Officer Name], our support officer kind of reminded us. So, I don't know whether you could have some kind of notification that would come up and remind…” | Silence |
| **The lesson observation form was difficult to find on the website, it was difficult to track lesson observation completion and some usability issues with completing out of internet range.** | **Usability**  Less than half of School Champions (n=6) were able to find the SAAFE lesson observation section on the website (46%). | **Difficult to find:**  “I believe it's good, but I would just, um, move the observations, um, to its own tab…”  “Um, you do have to scroll down a little bit on the page [to find the Lesson Observation Form], and it is like, quite a small link, which I know some staff at our school have had trouble, kind of, finding it.”  “Yes, is easy to find, once you have been there a few times.”  “I wouldn't say that I am confident in finding it um straight away, but I definitely can navigate it quite easily”  “I've used this [lesson observations] quite a few times already, so it was reasonably easy to find, I think because of that. The first time I looked for it, I thought it was actually in a little bit of a strange place and not very obvious, uh, but once you know where it is, it's kind of easy to remember where it is.”  **Difficult to track PE teachers completion**  “…you just kind of have to do a bit of a tally of how many observations one teacher has had.”  **Signal issues**  “Sometimes when we were out of mobile range or if this person didn't have a mobile um, it didn't quite, ah, work out.”  “Um, we also found on the online one you could get up on your phone. So that was really easy to do in professional learning.” | Agree |
| **Termly surveys were generally acceptable and completed.** | From 0 to 12 months, 24/24 School Champions completed all termly surveys. From 12 to 24 months, 21/24 School Champions completed all termly surveys (three schools missed 1 survey each in the period from 12 to 24 months of the programme). | “…the termly surveys, um, I mean this was handy to see where we were at but I suppose before I did the survey I kind of had an idea of what we did and didn't do well.” | Slightly agree |
| **The resources were acceptable and downloaded frequently.** | A total of 90 resources were downloaded from the website at least once by either an in-School Champion or a PE Teacher, equating to a total of 1007 downloads. | “Resources there are pretty good, but I would just continue to add more.”  “I am aware of all the main resources on the website. They're easily accessible and very useful.”  “I really love, um, this particular resource section. Um, yeah, look, I, I just can't say enough about, you know, what I love about it.”  “I loved the graphics and the images. I've really, I really liked working with the Physical Activity Policy template. I thought it looked really professional, um, um, yeah, I- I did really like ... those aspects.”  “Um, yeah well obviously I haven't really used this [pointing at the resources section] as much as I should've. I have used some of these [resources] though, but I must have just kind of forgotten where I'd got them from um, in the past.” | Agree |

**References**

1. Perski O, Blandford A, West R, Michie S. Conceptualising engagement with digital behaviour change interventions: a systematic review using principles from critical interpretive synthesis. Translational Behavioral Medicine. 2017;7(2):254-67.
